# Genomic, physiologic, and proteomic insights into metabolic versatility in *Roseobacter* clade bacteria isolated from deep-sea water

**DOI:** 10.1038/srep35528

**Published:** 2016-10-20

**Authors:** Kai Tang, Yujie Yang, Dan Lin, Shuhui Li, Wenchu Zhou, Yu Han, Keshao Liu, Nianzhi Jiao

**Affiliations:** 1 State Key Laboratory for Marine Environmental Science, Institute of Marine Microbes and Ecospheres, Xiamen University, Xiamen 361102, P. R. China

## Abstract

*Roseobacter* clade bacteria are ubiquitous in marine environments and now thought to be significant contributors to carbon and sulfur cycling. However, only a few strains of roseobacters have been isolated from the deep-sea water column and have not been thoroughly investigated. Here, we present the complete genomes of phylogentically closed related *Thiobacimonas profunda* JLT2016 and *Pelagibaca abyssi* JLT2014 isolated from deep-sea water of the Southeastern Pacific. The genome sequences showed that the two deep-sea roseobacters carry genes for versatile metabolisms with functional capabilities such as ribulose bisphosphate carboxylase-mediated carbon fixation and inorganic sulfur oxidation. Physiological and biochemical analysis showed that *T. profunda* JLT2016 was capable of autotrophy, heterotrophy, and mixotrophy accompanied by the production of exopolysaccharide. Heterotrophic carbon fixation via anaplerotic reactions contributed minimally to bacterial biomass. Comparative proteomics experiments showed a significantly up-regulated carbon fixation and inorganic sulfur oxidation associated proteins under chemolithotrophic conditions compared to heterotrophic conditions. Collectively, rosebacters show a high metabolic flexibility, suggesting a considerable capacity for adaptation to the marine environment.

*Roseobacter* clade bacteria (RCB) are one of the most abundant bacterioplanktonic groups in oceans worldwide, accounting for 10–25% and 2–15% of the total 16S ribosomal RNA microbial community in some surface ocean and sediment ecosystems, respectively^1^,^2^,^3^,^4^,^5^,^6^. All RCB cluster closely together within the *Rhodobacteraceae* family of *Alphaproteobacteria* and are widely distributed in various marine habitats from coastal regions to open oceans, surface water to sediments, and polar ice to tropical latitudes^1^,^2^,^3^,^4^,^5^,^6^. Moreover, RCB species are often found in symbiosis with marine algae^7^.

Marine RCB carry out critical carbon and sulfur biogeochemical transformations^6^,^8^,^9^,^10^. RCB are a metabolically diverse group of bacterioplankton with opportunitrophic lifestyles^11^. RCB may function to remineralize organic matter and recycle essential nutrients, especially in coastal environments and during phytoplankton blooms, where they often reach high abundance^12^,^13^. Certain species of RCB, termed aerobic anoxygenic phototrophic bacteria (AAPB) contain a photosynthetic gene cluster with the capability for photoheterotrophy^7^,^14^,^15^ and appear to play a unique role in the ocean’s carbon cycle^16^. AAPB have a non-autotrophic mechanism, anaplerotic CO_2_ assimilation, due to the absence of the key Calvin-Benson-Bassham (CBB) cycle enzymes ribulose bisphosphate carboxylase (RuBisCO) and phosphoribulokinase^14^,^17^. RCB have recently been reported to catabolize 2,3-dihydroxypropane-1-sulfonate as a molecular link in both the carbon and sulfur cycles^10^. Certain species of RCB have dimethylsulfoniopropionate (DMSP) assimilation genes into the climate-active gas dimethylsulfide^18^ and some have the Sox (sulfur oxidation proteins) multi-enzyme pathway for thiosulfate oxidation^6^. Thus, RCB play an important role in the ocean’s surface and sediment sulfur cycle^6^,^9^,^10^. However, previous studies have suggested that, unlike most known sulfur-oxidizing *Gammaproteobacteria*, RCB lack an autotrophic carbon fixation pathway^6^,^19^. In contrast, *Pelagibaca bermudensis* HTCC2601 isolated from surface water of the Sargasso Sea has genes that encode all CBB cycle enzymes^20^, indicating a potential autotrophic carbon fixation; however, there is no physiological evidence of this.

The deep ocean is the largest reservoir of organic carbon in the biosphere^21^. It harbors not only heterotrophic and autotrophic microbes but also mixotrophic microbes that significantly contribute to the carbon cycle of dark biosphere^21^,^22^,^23^. Cultivated isolates of RCB are currently classified into 72 genera and 222 species with a valid name, and only 8 species from 7 genera recently have been isolated from bathypelagic water (*Thiobacimonas profunda*^24^, *Pelagibaca abyssi*^25^, *Salipiger nanhaiensis*^26^, *Roseovarius halotolerans*^27^, *Roseovarius indicus*^28^, *Palleronia abyssalis*^29^, *Roseivivax marinus*^30^ and *Seohaeicola westpacificensis*^31^). More than 60 RCB genomes have been sequenced, however, none of deep-sea roseobacter strain sequencing genome is available. Therefore, our understanding of the metabolic potentials and functional traits of RCB in bathypelagic regions relative to representatives of RCB from other marine environments is limited. RCB species *T. profunda* JLT2016 and *P. abyssi* JLT2014 were obtained from the water column at depths of 2,571 m and 2,000 m, respectively, at two stations in the Southeastern Pacific, and were determined to be phylogenetically closely related^24^,^25^, which has provided unparalleled opportunities for investigation of the integrated mechanism of RCB metabolic adaptation to the marine environment. Here, the first look into the genome, physiology and proteome of deep-sea RCB substantially expands the current knowledge of marine RCB functional diversity and physiological activities.

## Results and Discussion

### General genome organization and content

**Figure 1:**
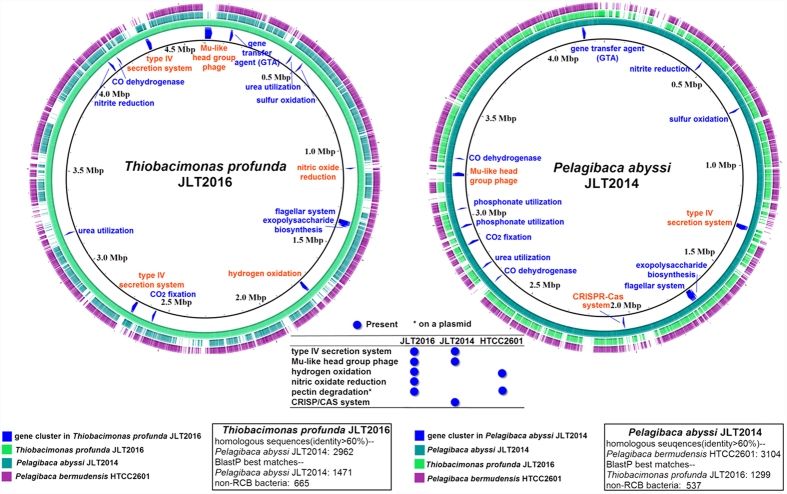
BRIG for multiple genome comparison. A composite genome comparison figure was generated using BRIG after performing a BLASTN analysis of *T. profunda* JLT2016 (left) and *P. abyssi* JLT2014 (right) as the reference genome. Each genome mapping to the reference is represented as a colored ring with a solid color representing greater than 60% sequence identity. Left: inner ring *T. profunda* JLT2016 (green), middle ring *P. abyssi* JLT2014 (light blue) outermost ring *Pelagibaca bermudensis* HTCC2601 (purple). Right: inner ring *P. abyssi* JLT2014 (light blue), middle ring *T. profunda* JLT2016 (green), outermost ring *P. bermudensis* HTCC2601 (purple). The major gene clusters in two deep-sea *Roseobacter* are labeled on the innermost ring with dark blue arcs. The gene clusters shared by three *Rosebacter* strains are highlighted with blue text, whereas gene clusters absent from one or two bacteria are highlighted with the orange text. The gene cluster information is provided in the table.

The general features of the *T. profunda* JLT2016 and *P. abyssi* JLT2014 genome are summarized in Table 1 and Fig. 1. Both genomes are comprised of a single circular chromosome and 8 plasmids. The *T. profunda* JLT2016 and *P. abyssi* JLT2014 chromosome size is 4.61 Mbp and 4.25 Mbp long, harboring a total of 4,539 and 4,204 predicted protein-coding sequences (CDSs) with a GC content of 67% and 66%, respectively. The plasmid size accounts for 15% and 19% of the total genome size, respectively. This is a moderate amount in known complete roseobacters genomes with plasmids, which possess between 2 and 11 plasmids in addition to their chromosome and the 2–33% plasmid borne in their genomes^32^. The average nucleotide sequence similarity between *T. profunda* JLT2016 and *P. abyssi* JLT2014 is 81%. Bidirectional BLAST analyses showed that approximately 3,000 genes exhibited >60% sequence identity among *T. profunda* JLT2016, *P. abyssi* JLT2014 and *P. bermudensis* HTCC2601, indicating identical or equivalent function. A total of 665 and 537 genes in *T. profunda* JLT2016 and *P. abyssi* JLT2014, respectively, shared a pangenome with other non-*Roseobacter* clade bacteria.

### Major functions shared by two deep-sea roseobacters

**Figure 2:**
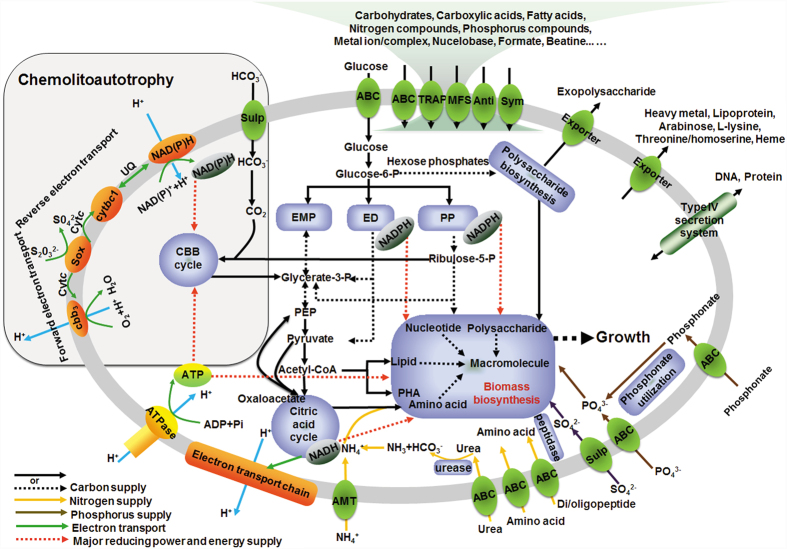
Metabolic pathways and transporter systems shared by *T. profunda* JLT2016 and *P. abyssi* JLT2014 based on functional genomics. The cytoplasmic membrane is depicted in grey. Different color arcs represent nutrient and energy metabolic pathways. Transporter systems are detailed in Supplementary Table S2. CBB, Calvin-Benson-Bassham cycle; Sox, the Sox multi-enzyme system; cbb3, cytochrome oxidase cbb3; cytbc1, cytochrome bc1 complex; Cytc, cytochrome c protein; UQ, ubiquinone; NAD(P)H, NADH (or NADPH)-quinone oxidoreductases; EMP, Embden-Meyerhof-Parnas; ED, Entner-Doudoroff; PP, pentose phosphate pathway; PEP, phosphoenolpyruvate; PHB, poly-3-hydroxybutyrate; ABC, ATP-binding cassette transporter systems; TRAP, tripartite ATP-independent periplasmic systems; MFS, major facilitator superfamily; Anti, antiporter; Sym, symporter; Sulp, sulfate or bicarbonate transporter; AMT, ammonium transporter.

The presence of gene sets in their chromosomes and plasmids assumed to be important for inhabiting marine environments within the genomes of the two strains were selected and summarized in Fig. 1 and Supplementary Table S1. *T. profunda* JLT2016 and *P. abyssi* JLT2014 shared major metabolic modules (Fig. 2).

#### Carbon fixation and sulfur oxidation

A striking genomic feature of the two strains is that they have the potential for CO_2_ fixation via the CBB cycle and inorganic sulfur oxidation, suggesting potential chemoautotrophic ability (Fig. 2). The complete CBB operons in *T. profunda* JLT2016 and *P. abyssi* JLT2014 showed high sequence identity and conserved gene structure (Supplementary Fig. S1). Phylogenetic analysis showed that RuBisCO in *T. profunda* JLT2016 and *P. abyssi* JLT2014 belong to the IC class RuBisCO (Supplementary Fig. S2). The Sox enzyme complex (*soxABXYZ*) for oxidation of thiosulfate (S_2_O_3_^2−^) to elemental sulfur was found in their genomes (Supplementary Fig. S1). Sox is proposed to produce energy and reducing power for carbon fixation via a reverse and forward electron transfer of sulfur oxidation (Fig. 2)^32^. The components of forward and reverse electron transport chains involved in sulfur oxidation include cytochrome c proteins, cytochrome bc1 complex and NADH (or NADPH)-quinone oxidoreductases (Fig. 2)^33^. Both genomes include terminal cytochrome oxidase genes for low (types aa3 and bo3) and high (types cbb3 and bd) affinity cytochrome c oxidases^34^, suggesting adaptation to growth in a wide range of oxygen concentrations. Other genes encode enzymes for the oxidation of reduced sulfur compounds, including sulfide quinone oxidoreductase and reverse dissimilatory sulfite reductase complex, which are responsible for the oxidation of sulfide and sulfite, respectively (Supplementary Table S1).

#### Central carbon metabolism

Genes for a complete tricarboxylic acid cycle (TCA), Embden-Meyerhof-Parnas (EMP, glycolysis), Entner-Doudoroff (ED) and pentose phosphate pathway (PP) were identified in both deep-sea roseobacter genomes (Fig. 2). Additionally, both genomes contained genes encoding enzymes in anaplerosis (pyruvate carboxylase) and cataplerosis (malic enzyme and phosphoenolpyruvate carboxykinase) that work together to ensure the appropriate balance of carbon flow into and out of the TCA cycle^35^. Moreover, malic enzyme may act as a major anaplerotic factor in *Roseobacter denitrificans* as well, which catalyzes the reversible oxidative decarboxylation of malate to pyruvate and CO_2_^17^.

#### Transporter systems

RCB contain the genes for a relatively high number of ATP- binding cassette systems (ABC transporters) and tripartite ATP-independent periplasmic (TRAP) systems compared to other bacterial groups^36^, which allow them to efficiently import organic matter. Consequently, they are adapted for dilute, heterogeneous growth substrates^12^. The highest number of transporter systems were ABC transporters (92 and 78 for *T. profunda* JLT2016 and *P. abyssi* JLT2014, respectively), followed by TRAP transporters (28 and 22), and major facilitator superfamily transporters (21 and 18) (Supplementary Table S1), accounting for 9.2% and 8.7% of the total CDSs of *T. profunda* JLT2016 and *P. abyssi* JLT2014, respectively. Predicted uptaking substrates for the complete transporter systems in *T. profunda* JLT2016 comprised a variety of carbohydrates, carboxylic acids, amino acids, peptides, metals and other nutrients (Supplementary Table S2). Both genomes were found to possess abundant exporters involved in the efflux of polysaccharide, heavy metals and metabolites (Supplementary Table S2). Transporter genes in plasmids of *T. profunda* JLT2016 and *P. abyssi* JLT2014 account for 15.1% and 29.3% of the total of transporter systems, respectively. Their plasmids carried genes encoding transporters such as TRAP systems, ABC transporters for amino acid, di/oligopeptide, ribose, glycerol-3-phosphate, and heavy metal efflux (Supplementary Table S2). The type IV secretion systems are present in chromosomes of *T. profunda* JLT2016 or *P. abyssi* JLT2014 (Supplementary Fig. S3), which are commonly used for both DNA and protein transfer between bacteria and between bacteria and host^37^.

#### Exopolysaccharide (EPS) biosynthesis

A number of genes associated with putative production and biosynthesis of exopolysaccharides present in *P. abyssi* JLT2014 and *T. profunda* JLT2016 (Supplementary Table S1) showed high sequence identity to those in an exopolysaccharide-producing RCB strain, *Salipiger mucosus* DSM16094, found in soil^38^.

Two roseobacter bacteria share other genetic potentials for likely an adaption to the marine environments characterized by low-nutrient (“*Nitrogen and phosphorus acquisition”, “Motility and chemotaxis”* in the Supplementary Material), low temperature and high-pressure conditions (“*Responses to stress”* in the Supplementary Material).

### Comparative genomics of marine roseobacters

*T. profunda* JLT2016 and *P. abyssi* JLT2014 show substantial genomic dissimilarity. For instance, *T. profunda* JLT2016 contains gene clusters associated with hydrogen oxidation and pectin utilization, whereas *P. abyssi* JLT2014 contains a CRISPR-Cas system (Fig. 1), indicating a high potential for individual adaptations (“*Distinct functional gene clusters for two deep-sea roseobacters*” in the Supplementary Material). The CRISPR-Cas system is also found in other RCB species, *Dinoroseobacter shibae* DFL-12. (Supplementary Table S3).

Similar to other many roseobacter genomes, two roseobacter genomes exhibit high plasticity with the rearrangement of large portions of its genome and gene acquisition, as evidenced by abundant transposases, and the presence of the conjugative plasmid, prophage-like elements and genomic islands (Supplementary Table S3, “*Mobile genetic elements”* and “*Genomic islands*” in the Supplementary Material). The genomic plasiticity might shape diverse metabolic adaptations to a wide diversity of ecological niches, as has been observed for RCB.

**Figure 3:**
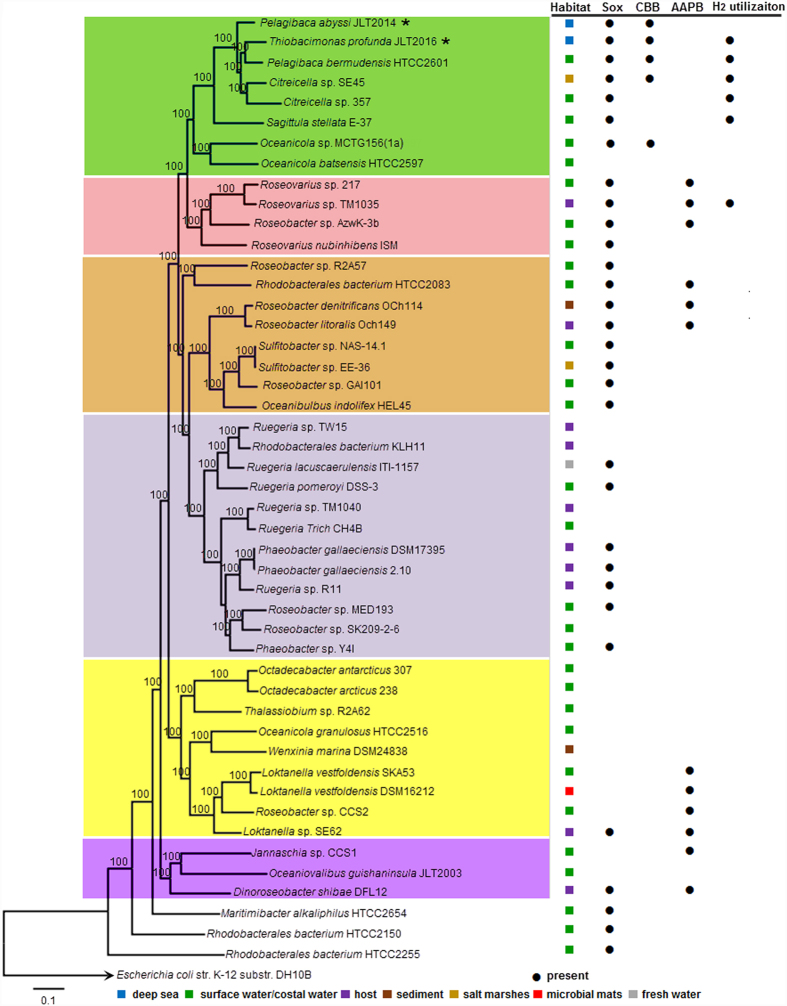
The genes cluster distributions marine RCB. The habits and gene clusters information in RCB are listed on the right. AAPB, photosynthetic gene cluster in aerobic anoxygenic phototrophic bacteria. Bootstrap values are shown on the branches of the phylogenetic tree. *Escherichia coli* K12 was used as an outgroup. The scale bar represents 10% sequence divergence.

Sulfur oxidation gene clusters (30/47) were found most frequently in roseobacter genomes, followed by photosynthetic genes (12/47) (Fig. 3). Only five marine roseobacters harbor genes encoding form IC Rubisco enzymes and other CBB genes (Supplementary Fig. S2). They belong to the same subclade including *Thiobacimonas, Pelagibaca, Oceanicola* and *Citreicella* (Fig. 3). Certain species of this subclade contained the H_2_ utilization gene cluster (Fig. 3). Most genes in the Sox, CBB and H_2_ gene clusters had high sequence identity (>70%) and conserved structures (Supplementary Figs S1 and S4). *T. profunda* JLT2016 and *P. abyssi* JLT2014 have CO dehydrogenase for aerobic CO oxidation but lack AAPB genes and DMSP utilization genes frequently found in roseobacters isolated from the surface water and alga-associated samples. Heterotrophy is essential for RCB success in the costal ocean whereas photoheterotrophy provide a survival advantage for AAPB in RCB in surface waters of the oligotrophic open ocean^12^,^16^. Pervious studies demonstrated that the *Gammaproteobacteria* (SUP05 clade and *Oceanospirillales*) and the *Deltaproteobacteria* (SAR324 clade) lineages that are ubiquitous in the dark oxygenated ocean are likely mixotrophs and have the potential for autotrophic CO2 fixation, coupled to the oxidation of reduced sulfur compounds^23^. Similarly, RCB bacteria within the *Alphabacteria* have the versatile metabolic potentials (heterotrophy, lithotrophy and authotrophy) that are beneficial for thriving at lower nutrients in the deep-sea than those in the surface water^39^.

### Growth experiments and physiological characteristics of *T. profunda* JLT2016

**Figure 4:**
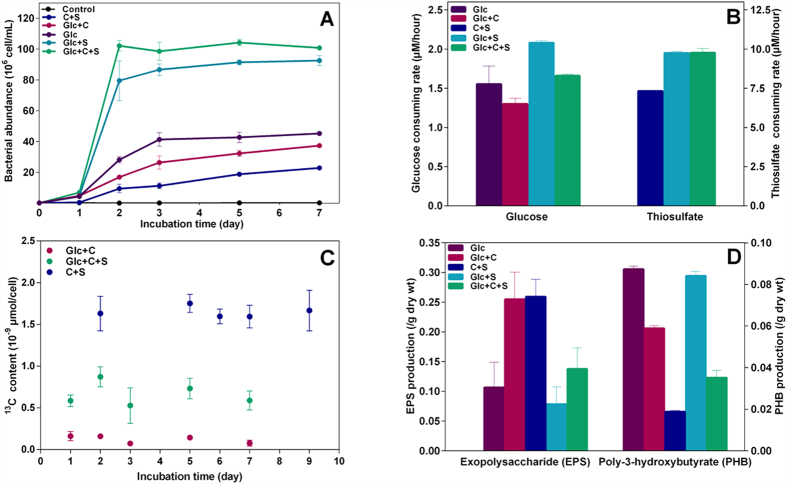
Laboratory batch experiments with *T. profunda* JLT2016 in an artificial seawater (ASW) base medium. **(A)** Growth of *T. profunda* JLT2016 incubated under different conditions. *T. profunda* JLT2016 was grown in an ASW base combined with different substrates. The bacteria were incubated in an ASW base without substrates as a control group. The concentrations of glucose (Glc), sodium bicarbonate (**C**) and thiosulfate (S) in the media were 100 μM, 2.5 mM and 1 mM, respectively. Autotrophic culture: C and S; mixotrophic culture: Glc, C and S; chemolithoheterotrophic culture: Glc and S; heterotrophic culture: Glc, or Glc and C. (**B**) The average rate of consumption of glucose and thiosulfate. (**C**) Tracking the content of ^13^C per cell during the growth. (**D**) Yield of EPS and PHB from *T. profunda* JLT2016 batch cultures at early stationary phase (dry wt, dry weight of cell). Error bars denote the SD of experimental replicates.

*T. profunda* JLT2016 could grow autotrophically (sodium bicarbonate as carbon source) and hetotropically (glucose as carbon source) by generating energy from chemolitotrophic sulfur oxidation (thiosulfate as an energy resource) (Fig. 4A–C). Moreover, *T. profunda* JLT2016 is able to grow with thiosulfate as an energy resource and using both sodium bicarbonate and glucose as carbon sources for growth (termed mixotrophy) (Fig. 4A–C). The new generations of sulfate concentrations under chemolithotrophic conditions are approximately 1.9-fold change relative to the added thiosulfate concentrations after exhaustion of thiosulfate, suggesting that bacteria could produce minimal amounts of elemental sulfur during thiosulfate oxidation, in contrast to the aerobic sulfur oxidizer *Gammaproteobacteria* SUP05 clade^40^. There was a significant difference in bacterial abudance under five culture conditions (one-way ANOVA, p < 0.05). The maximum final cell density during chemolithoheterotrophic growth (mean ± SD, 9.26 ± 0.31 cells × 10^7^/mL) was higher than that in heterotrophic growth with glucose (4.53 ± 0.08 cells × 10^7^/mL) (Fig. 4A), suggesting that heterotrophic cells could regulate the energy metabolism and biosynthesis after being supplied with thiosulfate as an alternative energy resource. *R. denitrificans* Och 114 has been reported to use anaplerotic pathways mainly via malic enzyme to fix 10% to 15% of protein carbon^17^. In contrast, ^13^C content in *T. profunda* JLT2016 when grown with glucose and NaH^13^CO_3_ is signifcantly less than that in autotrophic and mixotrophic cells (paired *t*-test, p < 0.001) (Fig. 4C), accounting for only 0.5–1.2% of the total carbon of cells. This finding suggests that the level of anaplerotic carbon fixation is low and bacterial growth relies primarily on heterotrophic metabolism (herein referred as heterotrophy). The ^13^C content in mixotrophic cells at 24 h was close to that in other growth phase, when cluture medium contained more than half of glucose added (Fig. 4B,C), indicating that autotrophic carbon fixation and heterotrophic metabolic pathways occur simultaneously. The average ^13^C content during the mixotrophic growth is only approximately 1/3 that during autotrophic growth (Fig. 4C), indicating that glucose was assimilated at the expense of chemolithotrophic energy, resulting in less carbon fixation.

The average cellular carbon (C) and nitrogen (N) ratio (4.09) of *T. profunda* JLT2016 is lower than the previously estimated for marine heterotrophic bacteria (4.31)^41^, but close to that estimated for marine dissolved organic carbon (4.13)^41^. Moreover, cellular C/N ratios varied very little among different trophic growth cells (Supplementary Fig. S5), suggesting that the trophic strategy does not have a great impact on cellular biochemical composition. Autotrophic growth cells yield the lowest amount of poly-3-hydroxybutyrate (PHB), which is a cellular carbon storage material (Fig. 4D). In contrast, autotrophic cells yield the highest amount of EPS, followed by cells grown on glucose and sodium bicarbonate. Specifically, their EPS accounted for 25.9% ± 2.9% and 25.5% ± 4.5% of dry cell weight, respectively, which was significantly higher than that observed for cells cultivated under other growth conditions (paired *t*-test, p < 0.05) (Fig. 4D). EPS biosynthesis consumed a great deal of energy and carbon, which may have been responsible for the relatively low cell densities in the presence of glucose and sodium bicarbonate mixture compared to those grown in the presence of glucose (Fig. 4A). EPS helps deep-sea bacteria endure extremes of temperature, salinity and nutrient availability^42^. Moreover, EPS can speed the rate of substance uptake and trap dissolved organic matter in the marine environment^42^. During *T. profunda* JLT2016 growth, microbial aggregates were detected in the liquid media and their aggregation ability was observed in by electron micrographs (Supplementary Fig. S6). Furthermore, aggregated bacterial cells surrounded by a continuous film of extracellular substances were observed by confocal laser scanning microscopy (Supplementary Fig. S7), indicating that *T. profunda* JLT2016 has biofilm-forming ability. Bacterial aggregates and biofilms enhance their competition and adaptation to the environment^43^. EPS may be involved in cell aggregation and biofim formation as previously suggested^43^.

### Comparative proteomics of *T. profunda* JLT2016

**Figure 5:**
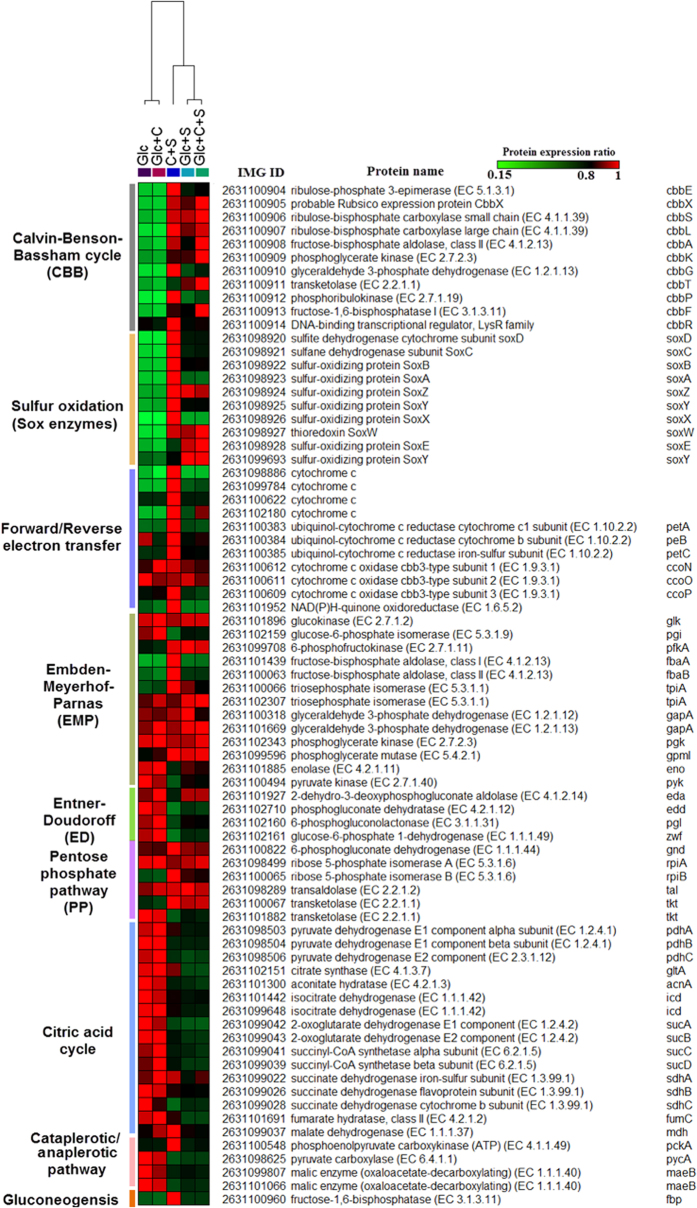
Proteomics analyses of CBB, inorganic sulfur oxidation and central metabolic pathways in different cultures. Each row represents the relative change of expressed proteins among samples. The value of normalized abundance of protein is assigned with a color relative to the maximum value among all comparisons of each protein. The colors represent the minimum (green), middle (black, 0.8) and maximum (red) values. Hierarchical clustering was performed based on the relative ratio in each protein using a Pearson correlation.

In total, 2,656 iTRAQ-labeled proteins, including 283 proteins on plasmids, were identified. Of these, 1,621 displayed significant differential expression between at least two growth conditions at early stationary phase (fold change >1.2 or <0.8 adopted in most iTRAQ studies based on accuracy and resolution of iTRAQ quantitation^44^,^45^. Overall, compared to heterotrophic growth, most of proteins in the CBB cycle and the Sox enzymes during autotrophic growth showed significantly higher abundance (Fig. 5). For example, the cbbL (ribulose-bisphosphate carboxylase large subunit) and soxB (sulfur-oxidizing protein soxB) proteins significantly increased by 3.8- and 4.2-fold, and 3.1- and 3.3-fold changes, respectively, in autotrophic cells relative to those in two heterotrophic cells grown with glucose, and glucose and sodium bicarbonate, whereas the mRNA abundance of the two genes relative to that of heterotrophic cells showed 8.2- and 5.5-fold changes, and 5.4- and 11.5-fold changes upon qRT-PCR, respectively (Supplementary Fig. S8). Moreover, cytochrome bc1 complexes, cytochrome c proteins, and a NAD(P)H-ubiquinone oxidoreductase (Fig. 5), which are predicted to be components of a reverse and forward electron transfer of sulfur oxidation, were up-regulated in autotrophic cells relative to those in heterotrophic growth. Cytochrome c oxidase type cbb3 in an electron transport was the only detected terminal oxidase under different conditions. Furthermore, most of proteins in the CBB cycle and the Sox enzymes in chemolithoheterotrophic and mixotrophic growth were present at higher levels than those in heterotrophic growth (Fig. 5). These findings combined with physiological evidence confirmed the existence of autotrophic and chemolithotrophic metabolic pathways in *T. profunda* JLT2016, as shown in Fig. 2.

When compared to other growth, key enzymes of central metabolisms were significantly up-regulated in heterotrophic growth including edd (phosphogluconate dehydratase) for ED pathways, and zwf (glucose-6-phosphate dehydrogenase) and pgl (6-phosphogluconolactonase) shared by ED pathway and PP pathway (Fig. 5). Most of enzymes in the TCA cycle in heterotrophic bacteria were also significantly upregulated than other growth (Fig. 5). ATP, NADPH, and NADH concentrations were higher in hetrotrophic cells than that in chemolithoheterotrophic and mixotrophic cells at early stationary phase (Supplementary Fig. S9, paried *t*-test, p < 0.01), thereby indicating an enhanced energy and reducing power production from central metabolism in heterotrophic cells. Bacterial growth is crucially dependent on protein synthesis and thus on the number of ribosomes^46^. Specifically, upregulated ribosomal protein expression was observed during chemolithoheterotrophic and mixotrophic growth (Supplementary Fig. S10), thereby indicating bacteria enhanced protein biosynthesis and futher increased cell abundance. Although chemolithoheterotrophic and mixotrophic growth required more energy for biosynthesis than heterotrophic growth, sulfur oxidation provides another ATP resource and reduces ATP production through oxidative phosphorylation, as indicated by the upregulation of the Sox enzymes and downregulation of the TCA cycle proteins (Fig. 5). These then gave rise to increased carbon flux toward biomolecules biosynthesis for cells under chemolithoheterotrophic and mixotrophic growth compared to heterotrophic growth. The expression of malic enzyme and pyruvate carboxylase was not affected by the switch from heterotrophic growth on glucose to heterotrophic growth on glucose and sodium carbonate (Fig. 5), indicating that anaplerotic carbon fixation in *T. profunda* JLT2016 did not appear to be important for bacterial growth. When compared to other types of growth, autotrophic growth upregulated phosphoenolpyruvate carboxykinase and the gluconeogenic enzyme fructose-1,6-bisphosphatase (fbp), which are required to bypass the irreversible step in glycolysis (Fig. 5). The increasing gluconeogenesis might lead to increased carbon flux to hexoses that are used in polysaccharides production. Most EPS biosynthesis proteins have relatively high abundance in autotrophic growth and heterotrophic growth in the presence of glucose and sodium bicarbonate mixture (Supplementary Fig. S10).

Transporter was the major functional category of detected proteins in all samples, accounting for 8.2% of the total. The most frequently observed transporters were ABC transporters (154/219) such as sugar, amino acid, and dipeptide/oligopeptide. The next most frequently observed transporter is TRAP (36/219). A SulP family transporter predicted to take up sodium bicarbonate was significantly up-regulated during autotrophic and mixotrophic growth, whereas glucose transporter was down-regulated in autotrophic growth (Supplementary Fig. S11). The transporters for ammonia, phosphate and sulfate were also detected (Supplementary Fig. S11). Bacteria expressed not only transporters for essential nutrients, but also transporters for other originally non-existent nutrients in media. For example, autotrophic growth cells have higher expression of a glycerol ABC transporter system in a plasmid (Supplementary Fig. S11). Even though, hydrogen gas, hydrogen sulfide and urea were not selected as growth substrates in this study, hydrogenase, sulfide quinone oxidoreductase and urease were detected in all samples (Supplementary Fig. S11). A previous study showed that approximately 50% of proteomes in several roseobacter strains represent an opportunistrophic gene pool, allowing rapid bacterial response to specific environmental changes^11^. Similarly, *T. profunda* JLT2016 not only has the bare minimum proteome for survival under different trophic conditions, but also follows an opportunitrophic lifestyle of maintaining broad functional potential to exploit spatially and temporally variable substrates as carbon and energy resources.

## Methods

### Genome sequencing

*T. profunda* JLT2016 and *P. abyssi* JLT2014 DNA were extracted using a TIANamp Bacteria DNA Kit (Tiangen, Beijing, China). The genome sequencing was performed at the Chinese National Human Genome Center at Shanghai.

The genome of *T. profunda* JLT2016 was sequenced using a massively parallel pyrosequencing technology (GS FLX+, Roche Diagnostics, Indianapolis, IN, USA). Briefly, 5 μg of sample DNA was sheared to 500–1000 bp by Covaris S2 (Covaris, Woburn, MA, USA) and purified using AMPure Beads (Beckman-Coulter, Fullerton, CA, USA). A DNA sequencing library was generated using a GS DNA Library Preparation Kit (Roche Diagnostics, USA) and a GS emPCR Kit (Roche Diagnostics, USA) for emPCR. A total of 248,283 reads counting up to 133.79 Mbp were obtained, covering 25.3-folds of the genome. Assembly was performed using Newbler (v2.7) and produced 136 contigs ranging from 500 bp to 383,719 bp. Relationships of the contigs were determined by multiplex PCR. Gaps were then filled in by sequencing PCR products using ABI 3730xl capillary sequencers (Applied Biosystems, Foster city, CA, USA). Finally, sequences were assembled using Phred, Phrap and Consed software packages (http://www.phrap.org/), and low quality regions of the genome were resequenced.

Whole genome sequencing of *P. abyssi* JLT2014 was accomplished using a hybrid approach, combining Illumina short read data with PacBio long read data^47^. Briefly, 1 μg of sample DNA was sheared to 300 bp by sonication with Covaris S2 (Covaris, USA). NEBNext Ultra™ DNA Library Prep Kit for Illumina (Illumina, San Diego, CA, USA) was used to construct the library, after which 10 ng of the sequencing library was used generate clusters in cBot using a TruSeq PE Cluster Kit (Illumina, USA), and finally sequenced by Illumina HiseqTM 2500 to generate 2 × 125 bp reads. A 5 μg sample DNA was then sheared to 10 Kb by Covaris g-TUBE (Covaris, USA). A PacBio SMRT bell™ Template Prep Kit (Pacific Biosciences, Menlo Park, CA, USA) was used to construct the library. The sequencing primers were annealed using a PacBio DNA/Polymerase Kit and polymerase combined to the SMRTbell templates (Pacific Biosciences, USA). PacBio sequencing data were generated using a PacBio RS-II instrument, C2 chemistry and one SMRT cell per genome (Pacific Biosciences, USA). A total of 685.67 M bp (post-filter) with an average length of 5664 bp were obtained. The genome sequences were de novo assembled by the HGAP2 program in the SMRT analysis server (v2.3). Illumina pair end reads were mapped to the assembled contigs to improve the accuracy of genome sequences.

### Bioinformatics analysis

The final assembled genomes were automatically annotated and analyzed through the IMG/ER (http://img.jgi.doe.gov). The comparison and visualization of multiple genomes was conducted with BRIG^48^. The concatenated conserved 70 single copy genes^49^ in the RCB were used for reconstruction of the maximum likelihood phylogenetic inference through the RAxML program (v7.4.2)^50^ under a JTT plus GAMMA model. Other bioinformtaic analysis methods were available in the Supplementary Material.

### Culture conditions

All reagents used in bacterial cultures were obtained from Sigma-Aldrich (St Louis, MO, USA) unless otherwise specified. Growth medium consisted of artificial seawater (ASW) base combined with substrates including glucose (Glc, 100 μM), NaHCO_3_ (C, 2.5 mM), and Na_2_S_2_O_3_ (S, 1 mM) in phosphate-buffered saline (PBS; pH 8.0) (autotrophic culture: C and S; mixotrophic culture: Glc, C and S; chemolithoheterotrophic culture: Glc and S; heterotrophic culture: Glc, or Glc and C). 2% cultures of *T. profunda* JLT2016 (2:100 dilution in PBS) in the expotential phase grown in the rich organic medium^51^ was used to inoculate the definite medium following 100-fold dilution by ASW to minimize the carryover of rich medium. Bacterial cells (approximately 1.8 × 10^5^ cells/mL) were then inoculated into 100-mL serum bottles (headspace: 75 mL volume) in an incubator (XMTE-8112, Sukun, China) with shaking (160 rpm) at 28 °C. Growth experiments were conducted in six replicates. pH and concentration of dissolved oxygen (DO) was monitored using a WTW-Multi3430 water quality checker (WTW, Weilheim, Germany), which revealed slight variations during culture growth. The ASW solution was prepared as follows: 20 g NaCl, 0.5 g MgSO_4_, 7H_2_O, 0.3 g KCl, 0.05 g, CaCl_2_, 2H_2_O, 0.3 g NH_4_Cl, 0.3 g K_2_HPO_4_ per L supplemented with 1.0 ml·L^−1^ trace elements and vitamins stock solution (trace elements: 3.15 g FeCl_3_·6H_2_O, 4.36 g EDTA-2Na·2H_2_O, 0.18 g MnCl_2_·4H_2_O, 9.8 mg CuSO_4_·5H_2_O, 6.3 mg Na_2_MoO_4_·2H_2_O, 22.0 mg ZnSO_4_·7H_2_O and 10.0 mg CoCl_2_·6H_2_O per L; vitamin: 0.02 g vitamin B_12_, 0.2 g niacin, 0.08 g biotin and 0.4 g thiamine per L).

### Flow cytometry measurements

Cells were stained with SYBR Green Ι (Invitrogen, Eugene, OR, USA) for 15 min, after which the abundance was monitored by EPICS ALTRA II flow cytometry (Beckman-Coulter, USA).

### Biochemical measurements

Glucose concentration in growth media was determined using a Glucose Colorimetric/Fluorometric Assay Kit (Biovision, Milpitas, CA, USA). Concentrations of NADH, NADPH and ATP were chemically tested with NAD^+^/NADH and NADP^+^/NADPH quantification kits (Biovision, USA) and an ATP Assay Kit (Abcam, Cambridge, MA, USA) according to the manufacturer’s instructions. Sulphate and thiosulfate in the culture medium measurements were carried out using a Dionex ICS-2500 ion chromatography system (Dionex, Sunnyvale, CA, USA). To analyze the elemental composition (C and N) samples were evaluated using a Vario EL Cube (Elementar Analysen Syetem GmbH, Germany). The PHB content in cells was determined by acid hydrolysis^52^ with HPLC using a Dionex ASI-100 autosampler injector (Dionex, USA) equipped with an Aminex HPX-87 H ion exchange organic acids column (300 × 7.8 mm) (BioRad, Hercules, CA, USA). Exopolysaccharides were determined by the phenol-sulfuric acid method^53^.

### Carbon isotope measurements

For analysis, ^13^C-labeled NaHCO_3_ (99% ^13^C, Cambridge Isotope Laboratories, MA, USA) was added into the culture medium as described above. Each sample was collected at different culture times using 0.3-μm glass fiber filters (GF-75, Advantec, Japan) that had been weighed and burned at 450 °C for 4 h. Sample filters were then freeze dried for about 2 days, after which they were weighed and packed into tin cans. The ^13^C content in cells was determined using a Flash EA 1112 Series elemental analyzer coupled with a ConfloIII interface to a Delta V Advantage isotope ratio mass spectrometer (Thermo Electron, San Jose, CA, USA). All defined samples were collected and analyzed in triplicate.

### iTRAQ-based quantitative proteomic analysis

Isobaric tags for relative and absolute quantitative (iTRAQ) MS/MS were used to analyze the proteome of *T. profunda* JLT2016 under different growth conditions as described above. The iTRAQ-MS/MS was performed at the Rearch Center for Proteome Analysis, Shanghai Institute for Biological Sciences, Chinese Academy of Sciences. Total proteins were extracted before iTRAQ analysis. Briefly, cell suspensions at early stationary phase (120 h for autotrophic cells, 48 h for mixotrophic cells and chemolithoheterotrophic cells, and 62 h for heterotrophic cells) were pelleted by centrifugation (7,000g, 4 °C, 15 min). After washing pellets with 0.2 M potassium phosphate buffer (pH 7.4), cells were added to STD buffer (4% SDS, 100 mM DTT, 150 mM Tris-HCl pH 8.0) and incubated in a water bath at 99 °C for 5 min. To improve cell lysis, samples were sonicated for 10 s, interval 15 s, and lasting for 2–3 min. After centrifugation (16,000*g*, 4 °C, 30 min), the supernatants were transferred into new tubes and prepared for subsequent analysis.

Protein digestion was performed according to a previously described FASP procedure^54^, after which the resulting peptide mixture was labeled using the 8-plex iTRAQ reagent according to the manufacturer’s instructions (Applied Biosystems, USA). iTRAQ labeled peptides were fractionated by strong cation exchange chromatography (SCX) using the AKTA Purifier system (GE Healthcare Amersham Biosciences, USA) as previously described^55^. The collected fractions were finally combined into 10 pools and desalted on SPE C18 Cartridges (Empore™ standard density bed I.D. 7 mm, volume 3 ml, Sigma-Aldrich, USA). Each fraction was concentrated by vacuum centrifugation and reconstituted in 40 μl of 0.1% (v/v) formic acid. Experiments were performed on a Q Exactive mass spectrometer (Thermo Electron, USA) coupled to an Easy nLC (Thermo Fisher Scientific, Waltham, MA, USA) according to a previous method^55^.

MS/MS spectra were searched using the MASCOT engine (Matrix Science, London, UK; v2.2) embedded into Proteome Discoverer 1.3 (Thermo Electron, USA) against *T. profunda* JLT2016 protein sequences and the decoy database. The MASCOT parameters were set as follows: peptide mass tolerance, 20 ppm, MS/MS tolerance, 0.1 Da, trypsin enzyme with up to 2 missed cleavages, fixed modification of iTRAQ 8-plex (K), iTRAQ 8-plex (N-term), variable modification of oxidation (M), and decoy database pattern of Reverse. A protein quantified with at least three peptides in experimental replicates was kept for further analysis (p < 0.05). Final ratios of protein quantification were then normalized by the median average protein quantification ratio for unequally mixed differently labeled samples.

## Additional Information

**Accession codes:** The complete genome sequence of *T. profunda* JLT2016 has been deposited in GenBank under the accession numbers CP014796 (chromosome), CP014797-CP014804 (plasmids pTPRO1- pTPRO8). The complete genome sequence of *P. abyssi* JLT2014 has been deposited in GenBank under the accession numbers CP015093 (chromosome), CP015091-CP015092 and CP015094-CP015097 (plasmids pPABY1- pPABY8). The complete genome sequences of *T. profunda* JLT2016 and *P. abyssi* JLT2014 have also been deposited at the through the Joint Genome Institute IMG/ER website (http://img.jgi.doe.gov) under the Genome ID 2630968259 and 2630968260, respectively.

**How to cite this article**: Tang, K. *et al*. Genomic, physiologic, and proteomic insights into metabolic versatility in *Roseobacter* clade bacteria isolated from deep-sea water. *Sci. Rep.* **6**, 35528; doi: 10.1038/srep35528 (2016).

## Supplementary Material

Supplementary Material

Supplementary Table S1

Supplementary Table S2

Supplementary Table S3

Supplementary Table S4

Supplementary Table S5

Supplementary Table S6

## Figures and Tables

**Table 1:** Summary of general genome features.

	*Thiobacimonas profunda* JLT2016	*Pelagibaca abyssi* JLT2014
Genome Size (Mbp)	5.42	5.26
Chromosome Size (Mbp)	4.61	4.25
Chromosome GC (%)	67	66
Chromosome CDS count	4,539	4,204
rRNA count	12	8
tRNA count	52	44
Plasmid count	8	8
Plasmid portion (%)*	15	19
Plasmid Size (Kbp)/GC (%)/CDS count	pTPRO1 (225.8/69/210)	pPABY1 (250.39/66/225)
	pTPRO2 (126.55/61/125)	pPABY2 (188.94/67/181)
	pTPRO3 (27.82/60/20)	pPABY3 (156.35/60/144)
	pTPRO4 (25.12/62/24)	pPABY4 (134.43/66/127)
	pTPRO5 (102.84/62/104)	pPABY5 (131.47/65/134)
	pTPRO6 (183.96/65/142)	pPABY6 (64.19/66/51)
	pTPRO7 (111.41/59/95)	pPABY7 (56.54/65/54)
	pTPRO8 (11.19/61/10)	pPABY8 (30.42/60/31)

^*^Plasmid portion is defined as the percent of the genome that is made up of a chromosome and plasmids.

## References

[b1] Selje N., Simon M. & Brinkhoff T. A newly discovered *Roseobacter* cluster in temperate and polar oceans. Nature 427, 445–448 (2004). 14749832 10.1038/nature02272

[b2] Buchan A., González J. M., Moran M. A. & Gonza M. Overview of the Marine *Roseobacter* Lineage. Appl. Environ. Microbiol. 71, 5665–5677 (2005). 16204474 10.1128/AEM.71.10.5665-5677.2005 PMC1265941

[b3] Moran M. A. . Ecological genomics of marine *Roseobacters*. Appl. Environ. Microbiol. 14, 4559–4569 (2007). 10.1128/AEM.02580-06 PMC1932822 17526795

[b4] Brinkhoff T., Giebel H.–A. & Simon M. Diversity, ecology, and genomics of the *Roseobacter* clade: a short overview. Arch. Microbiol. 189, 531–539 (2008). 18253713 10.1007/s00203-008-0353-y

[b5] Giebel H. . Distribution of *Roseobacter* RCA and SAR11 lineages in the North Sea and characteristics of an abundant RCA isolate. ISME J. 5, 8–19 (2010). 20596072 10.1038/ismej.2010.87 PMC3105675

[b6] Lenk S. . *Roseobacter* clade bacteria are abundant in coastal sediments and encode a novel combination of sulfur oxidation genes. ISME J. 6, 2178–2187 (2012). 22739490 10.1038/ismej.2012.66 PMC3504970

[b7] Wagner–do I. . The complete genome sequence of the algal symbiont *Dinoroseobacter shibae*: a hitchhiker’s guide to life in the sea. ISME J. 4, 61–77 (2010). 19741735 10.1038/ismej.2009.94

[b8] Wagner–do I. & Biebl H. Environmental biology of the marine *Roseobacter* lineage. Annu. Rev. Microbiol. 60, 255–280 (2006). 16719716 10.1146/annurev.micro.60.080805.142115

[b9] Howard E. C., Sun S., Biers E. J. & Moran M. A. Abundant and diverse bacteria involved in DMSP degradation in marine surface waters. Environ. Microbiol. 10, 2397–2410 (2008). 18510552 10.1111/j.1462-2920.2008.01665.x

[b10] Durham B. P. . Cryptic carbon and sulfur cycling between surface ocean plankton. Proc. Natl. Acad. Sci. USA 112, 453–457 (2015). 25548163 10.1073/pnas.1413137112 PMC4299198

[b11] Christie–Oleza J. A., Fernandez B., Nogales B., Bosch R. & Armengaud J. Proteomic insights into the lifestyle of an environmentally relevant marine bacterium. ISME J. 6, 124–135 (2012). 21776030 10.1038/ismej.2011.86 PMC3246242

[b12] Poretsky R. S., Sun S., Mou X. & Moran M. A. Transporter genes expressed by coastal bacterioplankton in response to dissolved organic carbon. Environ. Microbiol. 12, 616–627 (2010). 19930445 10.1111/j.1462-2920.2009.02102.x PMC2847192

[b13] Buchan A., Lecleir G. R., Gulvik C. A. & González J. M. Master recyclers: features and functions of bacteria associated with phytoplankton blooms. Nat. Rev. Microbiol. 12, 686–698 (2014). 25134618 10.1038/nrmicro3326

[b14] Swingley W. D. . The complete genome sequence of *Roseobacter denitrificans* reveals a mixotrophic rather than photosynthetic metabolism. J. Bacteriol. 189, 683–690 (2007). 17098896 10.1128/JB.01390-06 PMC1797316

[b15] Tang K., Zong R., Zhang F., Xiao N. & Jiao N. Characterization of the photosynthetic apparatus and proteome of *Roseobacter denitrificans*. Curr. Microbiol. 60, 124–133 (2010). 19826863 10.1007/s00284-009-9515-7

[b16] Jiao N. . Distinct distribution pattern of abundance and diversity of aerobic anoxygenic phototrophic bacteria in the global ocean. Environ. Microbiol. 9, 3091–3099 (2007). 17991036 10.1111/j.1462-2920.2007.01419.x

[b17] Tang K. H., Feng X., Tang Y. J. & Blankenship R. E. Carbohydrate metabolism and carbon fixation in *Roseobacter denitrificans* OCh114. PLoS One 4, e7233 (2009). 19794911 10.1371/journal.pone.0007233 PMC2749216

[b18] Bürgmann H. . Transcriptional response of *Silicibacter pomeroyi* DSS–3 to dimethylsulfoniopropionate (DMSP). Environ. Microbiol. 9, 2742–2755 (2007). 17922758 10.1111/j.1462-2920.2007.01386.x

[b19] Lenk S. . Novel groups of *Gammaproteobacteria* catalyse sulfur oxidation and carbon fixation in a coastal, intertidal sediment. Environ. Microbiol. 13, 758–774 (2011). 21134098 10.1111/j.1462-2920.2010.02380.x

[b20] Thrash J. C. . Genome sequences of *Pelagibaca bermudensis* HTCC2601^T^ and *Maritimibacter alkaliphilus* HTCC2654^T^, the type strains of two Marine *Roseobacter* genera. J. Bacteriol. 192, 5552–5553 (2010). 20729358 10.1128/JB.00873-10 PMC2950497

[b21] Follett C. L., Repeta D. J., Rothman D. H., Xu L. & Santinelli C. Hidden cycle of dissolved organic carbon in the deep ocean. Proc. Natl. Acad. Sci. USA 111, 16706–16711 (2014). 25385632 10.1073/pnas.1407445111 PMC4250131

[b22] Reinthaler T., van Aken H. M. & Herndl G. J. Major contribution of autotrophy to microbial carbon cycling in the deep North Atlantic’s interior. Deep Sea Res. Part II Top Stud. Oceanogr 57, 1572–1580 (2010).

[b23] Swan B. K. . Potential for chemolithoautotrophy among ubiquitous bacteria lineages in the dark ocean. Science 333, 1296–1300 (2011). 21885783 10.1126/science.1203690

[b24] Li S., Tang K., Liu K. & Jiao N. *Thiobacimonas profunda* gen. nov., sp. nov., a member of the family *Rhodobacteraceae* isolated from deep–sea water. Int. J. Syst. Evol. Microbiol. 65, 359–364 (2015). 25355706 10.1099/ijs.0.066449-0

[b25] Lin Y. . *Pelagibaca abyssi* sp. nov., of the family *Rhodobacteraceae*, isolated from deep–sea water. Antonie van Leeuwenhoek, Int. J. Gen. Mol. Microbiol. 106, 507–513 (2014). 10.1007/s10482-014-0219-z 24969947

[b26] Dai X., Shi X., Gao X., Liang J. & Zhang X. H. *Salipiger nanhaiensis* sp. nov., a bacterium isolated from deep sea water. Int. J. Syst. Evol. Microbiol. 65, 1122–1126 (2015). 25589735 10.1099/ijs.0.000066

[b27] Oh Y. S. . *Roseovarius halotolerans* sp. nov., isolated from deep seawater. Int. J. Syst. Evol. Microbiol. 59, 2718–2723 (2009). 19625435 10.1099/ijs.0.002576-0

[b28] Lai Q. . *Roseovarius indicus* sp. nov., isolated from deep–sea water of the Indian Ocean. Int. J. Syst. Evol. Microbiol. 61, 2040–2044 (2011). 20851912 10.1099/ijs.0.023168-0

[b29] Albuquerque L. . *Palleronia abyssalis* sp. nov., isolated from the deep Mediterranean Sea and the emended description of the genus *Palleronia* and of the species *Palleronia marisminoris*. Antonie van Leeuwenhoek, Int. J. Gen. Mol. Microbiol. 107, 633–642 (2015). 10.1007/s10482-014-0358-2 25524421

[b30] Dai X., Shi X., Gao X., Liu J. & Zhang X. H. *Roseivivax marinus* sp. nov., isolated from deep water. Int. J. Syst. Evol. Microbiol. 64, 2540–2544 (2014). 24812358 10.1099/ijs.0.062760-0

[b31] Xian S. . *Seohaeicola westpacificensis* sp. nov., a novel, member of genera *Seohaeicola* isolated from deep West Pacific sea water. Curr. Microbiol. 69, 32–36 (2014). 24585075 10.1007/s00284-014-0548-1

[b32] Pradella S., Päuker O. & Petersen J. Genome organisation of the marine *Roseobacter* clade member *Marinovum algicola*. Arch. Microbiol. 192, 115–126 (2010). 20039020 10.1007/s00203-009-0535-2

[b33] Scott K. M. . The genome of deep–sea vent chemolithoautotroph *Thiomicrospira crunogena* XCL–2. PLoS Biol. 4, e383 (2006). 17105352 10.1371/journal.pbio.0040383 PMC1635747

[b34] Morris R. L. & Schmidt T. M. Shallow breathing: bacterial life at low O(2) Nat. Rev. Microbiol. 11, 205–212 (2013). 23411864 10.1038/nrmicro2970 PMC3969821

[b35] Owen O. E., Kalhan S. C. & Hanson R. W. The key role of anaplerosis and cataplerosis for citric acid cycle function. J. Biol. Chem. 277, 30409–30412 (2002). 12087111 10.1074/jbc.R200006200

[b36] Tang K., Jiao N., Liu K., Zhang Y. & Li S. Distribution and functions of TonB–dependent transporters in marine bacteria and environments: implications for dissolved organic matter utilization. PLoS One 7, e41204 (2012). 22829928 10.1371/journal.pone.0041204 PMC3400609

[b37] Wallden K., Rivera–Calzada A. & Waksman G. Type IV secretion systems: Versatility and diversity in function. Cell Microbiol. 12, 1203–1212 (2010). 20642798 10.1111/j.1462-5822.2010.01499.x PMC3070162

[b38] Riedel T. . Genome sequence of the exopolysaccharide–producing *Salipiger mucosus* type strain (DSM 16094^T^), a moderately halophilic member of the *Roseobacter* clade. Stand. Genomic Sci. 9, 1331–1343 (2014). 25197501 10.4056/sigs.4909790 PMC4148975

[b39] Jiao N. . Microbial production of recalcitrant dissolved organic matter: long–term carbon storage in the global ocean. Nat. Rev. Microbiol. 8, 593–599 (2010). 20601964 10.1038/nrmicro2386

[b40] Marshall K. T. & Morris R. M. Isolation of an aerobic sulfur oxidizer from the SUP05/Arctic96BD–19 clade. ISME J. 7, 452–455 (2013). 22875135 10.1038/ismej.2012.78 PMC3554405

[b41] Suttle C. A. Marine viruses–major players in the global ecosystem. Nat. Rev. Microbiol. 5, 801–812 (2007). 17853907 10.1038/nrmicro1750

[b42] Poli A., Anzelmo G. & Nicolaus B. Bacterial exopolysaccharides from extreme marine habitats: Production, characterization and biological activities. Mar. Drugs 8, 1779–1802 (2010). 20631870 10.3390/md8061779 PMC2901825

[b43] Flemming H. C. & Wingender J. The biofilm matrix. Nat. Rev. Microbiol. 8, 623–633 (2010). 20676145 10.1038/nrmicro2415

[b44] Bantscheff M. . Robust and sensitive iTRAQ quantification on an LTQ Orbitrap mass spectrometer. Mol. Cell Proteomics 7, 1702–1713 (2008). 18511480 10.1074/mcp.M800029-MCP200 PMC2556025

[b45] Karp N. A. . Addressing accuracy and precision issues in iTRAQ quantitation. Mol. Cell Proteomics 9, 1885–1897 (2010). 20382981 10.1074/mcp.M900628-MCP200 PMC2938101

[b46] Klumpp S., Scott M., Pedersen S. & Hwa T. Molecular crowding limits translation and cell growth. Proc. Nat.l Acad. Sci. USA 110, 16754–16759 (2013). 10.1073/pnas.1310377110 PMC3801028 24082144

[b47] Koren S. . Hybrid error correction and de novo assembly of single–molecule sequencing reads. Nat. Biotechnol. 30, 693–700 (2012). 22750884 10.1038/nbt.2280 PMC3707490

[b48] Alikhan N.–F., Petty N. K., Ben Zakour N. L. & Beatson S. A. BLAST Ring Image Generator (BRIG): simple prokaryote genome comparisons. BMC Genomics 12, 402 (2011). 21824423 10.1186/1471-2164-12-402 PMC3163573

[b49] Newton R. J. . Genome characteristics of a generalist marine bacterial lineage. ISME J. 4, 784–798 (2010). 20072162 10.1038/ismej.2009.150

[b50] Stamatakis A. RAxML–VI–HPC: Maximum likelihood–based phylogenetic analyses with thousands of taxa and mixed models. Bioinformatics 22, 2688–2690 (2006). 16928733 10.1093/bioinformatics/btl446

[b51] Yurkov V. V., Krieger S., Stackebrandt E. & Beatty J. T. *Citromicrobium bathymonarium*, a novel aeorobic bacterium isolated from deep sea hydrothermal vent plume waters that contains photosynthetic pigment–protein complexes. J. Bacteriol. 181, 4517–4525 (1999). 10419948 10.1128/jb.181.15.4517-4525.1999 PMC103581

[b52] Karr D. B., Waters J. K. & Emerich D. W. Analysis of poly–β–hydroxybutyrate in *Rhizobium japonicum* bacteroids by ion–exclusion high–pressure liquid chromatography and UV detection. Appl. Environ. Microbiol. 46, 1339–1344 (1983). 16346443 10.1128/aem.46.6.1339-1344.1983 PMC239573

[b53] DuBois M., Gilles K. A., Hamilton J. K., Rebers P. A. & Smith F. Colorimetric method for determination of sugars and related substances. Anal. Chem. 28, 350–356 (1956).

[b54] Zougman A., Nagaraj N. & Mann M. Universal sample preparation method for proteome analysis. Nat. Methods 6, 3–7 (2009). 19377485 10.1038/nmeth.1322

[b55] Wang L. . Dynamics of chloroplast proteome in salt–stressed mangrove Kandelia candel (L.) druce. J. Proteome. Res. 12, 5124–5136 (2013). 24070322 10.1021/pr4006469

