# Supplementary material for: Genomic, physiologic, and proteomic insights into metabolic versatility in Roseobacter clade bacteria isolated from deep-sea water
Source: Sci Rep. 2016 Oct 20;6:35528. doi: 10.1038/srep35528 (PMC5071866; doi:10.1038/srep35528)
Supplement: Supplementary Material [file srep35528-s1.pdf]

Supplementary material

Genomic, physiologic, and proteomic insights into metabolic versatility in *Roseobacter* clade bacteria isolated from deep-sea water

Kai Tang*, Yujie Yang, Dan Lin, Shuhui Li, Wenchu Zhou, Yu Han, Keshao Liu, Nianzhi Jiao*

State Key Laboratory for Marine Environmental Science, Institute of Marine Microbes and Ecospheres, Xiamen University, Xiamen 361102, P. R. China

* Corresponding: tangkai@xmu.edu.cn and jiao@xmu.edu.cn

Results and discussion

Other functions shared by two deep-sea roseobacters.

Nitrogen and phosphorus acquisition. The genomes contained two transporters for ammonia (Supplementary Table S2). *T. profunda* JLT2016 and *P. abyssi* JLT2014 genomes harbor 77 and 88 proteases, respectively, as well as urease, indicating a pronounced specialization for the degradation of proteins, peptides and urea. The genomes not only have phosphate transporters for taking up phosphorus, but also alkaline phosphatase and phosphatases (phn) for degradation of dissolved organic phosphate. The *phn* genes of *T. profunda* JLT2016 are located on a plasmid (pTPRO01), while two sets of *phn* genes are found in the chromosome of *P. abyssi* JLT2014 (Supplementary Fig. S12). However, one pair of *phn* genes in *T. profunda* JLT2016 and *P. abyssi* JLT2014 showed a moderate level of sequence identity, which was within the range 53%–85%, and their gene structure is distinguished by the location of phosphonate ABC transporters (Supplementary Fig. S12), supporting the view that plasmid-mediated horizontal gene transfer (HGT) may involve the evolution of phosphonate degradation in bacteria[1].

Motility and chemotaxis. The genomes of both strains contain one large gene set encoding a potential lateral flagellum system for swarming (Supplementary Table S1). Furthermore, one potential lateral flagellum gene is located in a plasmid of *P. abyssi* JLT2014 (pPABY05) (Supplementary Table S1), which shows only low identity to the majority of flagellar genes in the chromosomes. Bacterial flagellar chemotaxis and methyl-accepting chemotaxis proteins were also identified (Supplementary Table S1). Overall, these findings indicate that these organisms may use chemotaxis and flagellum systems to facilitate their movement towards nutrient rich zones[2].

Responses to stress. Both genomes contain putative transporters for osmolytes taurine, glycine betaine, putrescine, and choline (Supplementary Table S2), as well as ectoine and betaine biosynthetic pathways to cope with osmotic stress (Supplementary Table

S1). The presence of cold shock proteins and antifreeze proteins could enable bacteria to be active under the cold deep-sea conditions. Both have extensive DNA repair systems to cope with the extreme conditions that have potential deleterious effects on the genomes (Supplementary Table S1).

Distinct functional gene clusters for two deep-sea roseobacters.

Hydrogen (H_2) utilization. One gene locus in the *T. profunda* JLT2016 genome has a cluster of genes encoding a membrane-bound nickel-iron uptake hydrogenase (*hupS*, *hupL*, and *hupC*) and multiple accessory proteins for the synthesis and assembly of this enzyme (Supplementary Fig. S4). In contrast to *P. abyssi* JLT2014, *P. bermudensis* HTCC2601 has a H_2 utilization gene cluster (Supplementary Fig. S4). An arrangement of the H_2 utilization gene cluster in *Rhodobacter sphaeroides* ATCC17025 was identical to that in *T. profunda* JLT2016; however, the *hupE* gene was not found in other roseobacters (Supplementary Fig. S4). Hydrogen utilization is typically mediated by uptake hydrogenases that catalyze the oxidation of hydrogen, allowing bacterial autotrophic growth on a hydrogen gas-based inorganic energy source[3].

CRISPR-Cas system. The presence of clustered regularly interspaced palindromic repeat (CRISPR) arrays and their associated Cas genes in *P. abyssi* JLT2014 form a system possibly involved in bacterial defense against phages or plasmids (Supplementary Fig. S13). The CRISPR-Cas system in *P. abyssi* JLT2014 belonged to type IE Cas protein sequences and showed closer similarities to the one in *Rhodovulum* sp. NI22 (Supplementary Fig. S13) than in *Dinoroseobacter shibae* DFL-12[4]. Furthermore, the consensus repeat sequences only have a one base difference between *P. abyssi* JLT2014 and *Rhodovulum* sp. NI22 (Supplementary Fig. S13), indicating their CRISPR-Cas system could originate from a common ancestor. However, CRISPR sequences revealed no similarity between strains with regards to

the numbers of repeats and spacer sequences, indicating that the histories of phage infection are different.

Pectin utilization. T. profunda JLT2016 and *P. abyssi* JLT2014 have 34 and 30 genes encoding glycoside hydrolases (GH), respectively. Two glycoside hydrolases for pectin degradation are organized in a polysaccharide utilization locus (PULs) in a plasmid (pTPRO01) of *T. profunda* JLT2016, where they are clustered with an oligogalacturonide transporter and a TRAP transporter system for the pectin degradation product galacturonate, as well as genes associated with hexuronate metabolism (Supplementary Fig. 14SA). Pectin degradation experiments confirmed this additional metabolic ability introduced by a plasmid (pTPRO01) of *T. profunda* JLT2016, as shown in Supplementary Fig. 14SB. *P. bermudensis* HTCC2601 and *S. mucosus* DSM16094 also contained similar PULs (Supplementary Fig. 14SA). Pectin PULs in roseobacters were similar to those in the members of the order *Rhizobiales* (Supplementary Fig. 14SA), suggesting that their gene clusters might have diverged from a common ancestor.

Mobile genetic elements. A large inversion and translocation forming a non-consecutive "X" pattern was revealed in the alignment of the chromosomes of the two species, suggesting that they share a high amount of genetic material, but their chromosomes have been subject to frequent rearrangements and events of horizontal gene transfer (Supplementary Fig. S15). Phages, gene transfer agents (GTA), plasmids and transposons can act as major vectors of HGT among diverse bacterial populations. Unusually large numbers of transposase-related genes (80 and 71 for *T. profunda* JLT2016 and *P. abyssi* JLT2014, respectively) were found in their genomes. These genes indirectly facilitate chromosomal rearrangements via homologous recombination events. Mobile genetic elements such as transposases, integrases and recombinases are found on their plasmids, and they have many identical copies on their chromosomes (Supplementary Fig. S16), suggesting that homologous recombination events occur frequently between plasmids and chromosomes. A

plasmid of *T. profunda* JLT2016 or *P. abyssi* JLT2014 coding for its own set of type IV secretion system, the coupling proteins (VirD4) and a relaxase VirD2 probably represent a conjugative extrachromosomal element that facilitates the occurrence of genetic exchange between distantly related microbes (Supplementary Fig. S3)[5]. Both bacteria have a predicted GTA (Supplementary Table S1). *T. profunda* JLT2016 and *P. abyssi* JLT2014 carry two and five prophage-like elements, respectively (Supplementary Table S4), in which their Mu-like head group phages have been induced successfully (Tang, et al. manuscript in preparation). In contrast, *P. bermudensis* HTCC2601 has no mobilizable extrachromosomal element, which is likely due to the absence of a type IV secretion system and only several phage-related sequences in the genome, indicating there is a comparatively large potential for genetic mobility and versatility encoded in the deep-sea roseobacters.

Genomic islands. HGT involves the incorporation of genetic elements, perhaps directly integrated into bacterial chromosomes where they form genomic islands (GIs)[6]. A total of 24 and 20 GIs were identified in chromosomes of *T. profunda* JLT2016 and *P. abyssi* JLT2014, respectively (Supplementary Fig. S17, Table S5 and Table S6). Their GIs are enriched in transposases and transporters and have type IV secretion system genes (Supplementary Fig. S17). The GIs in *T. profunda* JLT2016 contained more integrase and recombinase than those in *P. abyssi* JLT2014 (Supplementary Fig. S17). Genes encoding the flagellar system were present in the GI of *T. profunda* JLT2016, while a CRISPR-Cas system gene cluster was present in the GI of *P. abyssi* JLT2014 (Supplementary Fig. S17 and Table S6), suggesting that they could have been horizontally transferred.

Methods

Bioinformatics analysis. A maximum-likelihood tree of *cbbL* was computed using MEGA (v6.0)[7] with the LG+F model plus a gamma distribution with four categories. Multiple-genome alignment was conducted via Mauve[8]. The prophage identification

tool PHAge Search Tool (PHAST)[9] was used to predict regions containing prophage-like elements and then manually check them. Clustering of sequences into homologous families was performed using SiLiX (v1.2.8)[10] with a minimum identity threshold of 90% and default values for the remaining parameters. The CRISPRfinder program[11] (http://crispr.u-psud.fr/crispr) was used to identify CRISPR-like arrays in the genome. The genomic islands were predicted with the Islandview3 software[12].

Pectin utilization. *T. profunda* JLT2016 and *P. bermudensis* HTCC2601 were cultivated in pectin medium, which was prepared using artificial seawater, per L, 15 g agar, 23 g sea salt and 2 g apple pectin (>99%) supplemented with trace elements and vitamins described above and the medium was adjusted to pH 7.6–7.8 with 1% HCl. The same medium without pectin served as a blank. The culture was cultivated in an incubator (XMTE-8112, Sukun, China) at 28°C for 2–3 days to observe growth.

Biofilm development. Biofilms of *T. profunda* JLT2016 were grown in marine broth 2216 culture (Difco, Detroit, MI, USA) in 90-mm coverslip-bottom cultureware according to a previous method[13]. Two days later, media was removed, and the biofilm was washed with 10 mM phosphate-buffered saline solution to release unattached cells. Next, the biofilm was stained with BOBO-3 (Invitrogen, Eugene, OR, USA) and Calcofluor (Sigma-Aldrich, USA) for an hour in the dark. Following incubation, the biofilm was visualized by Carl Zeiss LSM 780 confocal laser scanning microscopy (Carl Zeiss MicroImaging GmbH, Göttingen, Germany). Excitation/emission wavelengths of the fluorophores were 400/490 nm and 570/602 nm, respectively.

Transmission electron microscopy. One drop of bacteria suspension was adsorbed onto a Formvar/carbon-coated 200 mesh copper grid for 10 min, then negatively stained with 20 g/L phosphotungstic acid in the dark for 30 s. After drying for 30 min, the grid was examined using a JEM-2100 at 120 KeV (JEOL, Tokyo, Janpan).

RT-PCR RNA extraction and quantitative real-time PCR (qRT-PCR). Cells of T. profunda JLT2016 for RNA isolation and purification were harvested at early

stationary phase under different culture conditions. RNA was extracted using TRIzol reagent (Invitrogen, USA) according to the manufacturer's protocols and quantified using a Nanodrop 2000 spectrophotometer (Thermo Fisher Scientific, USA). cDNA of each RNA sample was synthesized using a GoTaq two-step Real-Time PCR system (Promega, Madson, WI, USA). qRT-PCR was performed on a CFX96TM Real-Time System (BioRad, USA). Gene primers (cbbL-F, GATCCAGTCGATGGCGAAATG, cbbL-R, GATGACGCGGAAGCTGACAC, soxB-F, GACATGGTCAACGTGATGAACG, soxB-R,GAAGAACTTGTAGGGCTTGAAGAGC) were designed with the Primer Express software (Applied Biosystems, USA) and analyzed using OligoAnalyzer (v3.0). A 25 µl final volume of GoTaq qPCR 2× Master Mix (Promega, USA) was used to amplify DNA. The program for amplification was as follows, an initial hot-start activation step (2 min at 95°C) and denaturation step (15 sec at 95°C), followed by 40 cycles of 15 s at 95°C and 60 s at 64.3°C.

References

1. Huang, J., Su, Z. & Xu, Y. The evolution of microbial phosphonate degradative pathways. *J. Mol. Evol.* **61,** 682–690 (2005).

2. Stocker, R. & Seymour, J. R. Ecology and physics of bacterial chemotaxis in the ocean. *Microbiol. Mol. Biol. Rev.* **76,** 792–812 (2012).

3. Wrighton, K. C., *et al.* Fermentation, hydrogen, and sulfur metabolism in multiple uncultivated bacterial phyla. *Science* **337,** 1661–1665 (2012).

4. Wagner–do, I., *et al.* The complete genome sequence of the algal symbiont *Dinoroseobacter shibae* : a hitchhiker's guide to life in the sea. *ISME J.* **4,** 61–77 (2010).

5. Shintani, M., Sanchez, Z. K. & Kimbara, K. Genomics of microbial plasmids: Classification and identification based on replication and transfer systems and host taxonomy. *Front. Microbiol.* **6,** DOI=10.3389/fmicb.2015.00242 (2015).

7. Tamura, K., Stecher, G., Peterson, D., Filipski, A. & Kumar, S. MEGA6: Molecular evolutionary genetics analysis version 6.0. *Mol. Biol. Evol.* **30,** 2725–2729 (2013).

8. Darling, A. C. E., Mau, B., Blattner, F. R. & Perna, N. T. Mauve: multiple alignment of conserved genomic sequence with rearrangements. *Genome Res.* **14,** 1394–1403 (2009).

9. Zhou, Y., Liang, Y., Lynch, K. H., Dennis, J. J. & Wishart, D. S. PHAST: a fast phage search tool. *Nucleic Acids Res.* **39,** W347–352 (2011).

10. Miele, V., Penel, S. & Duret, L. Ultra–fast sequence clustering from similarity networks with SiLiX. *BMC Bioinformatics* **12,** 116 (2011).

11. Grissa, I., Vergnaud, G. & Pourcel, C. CRISPRcompar: a website to compare clustered regularly interspaced short palindromic repeats. *Nucleic Acids Res.* **36,** 52–57 (2008).

12. Dhillon, B. K., *et al.* IslandViewer3: more flexible, interactive genomic island discovery, visualization and analysis. *Nucleic Acids Res.* **43,** W104–108 (2015).

13. Charlebois, A., Jacques, M. & Archambault, M. Biofilm formation of *Clostridium perfringens* and its exposure to low–dose antimicrobials. *Front. Microbiol.* **5,** DOI=10.3389/fmicb.2014.00183 (2014).

Tables

Table S1. Quantitative comparative analysis of key enzymes identified in *T. profunda* JLT2016 and *P. abyssi* JLT2014.

Table S2. Predicted transporter systems and their substrates in *T. profunda* JLT2016 and *P. abyssi* JLT2014.

Table S3. Predicted CRISRPS-Cas sytem, mobile genomic elements, and genomic islands in the complete genomes of roseobacters.

Table S4. Predicted prophages in *T. profunda* JLT2016 and *P. abyssi* JLT2014.

Table S5. Genomic context of predicted genomic islands of *T. profunda* JLT2016.

Table S6. Genomic context of predicted genomic islands of *P. abyssi* JLT2014.

Figure legends

Fig. S1. CBB and Sox gene clusters in roseobacters. Gene descriptions of *T. profunda* JLT2016 are listed along the row. The numbers in the table represent sequence identity (%) between *T. profunda* JLT2016 and other bacteria at the amino acid level.

Fig. S2. Phylogenetic tree based on ribulose bisphosphate carboxylase large subunit (cbbL) genes amino acid sequence comparisons constructed using the maximum-likelihood method in MEGA6. The classes of RubisCO are listed on the right.

Fig. S3. Type IV secretion system gene clusters in two deep-sea roseobacter bacteria and other bacteria.

Fig. S4. The uptake hydrogenase gene cluster in *T. profunda* JLT2016 and other bacteria.

Fig. S5. Determination of cellular C/N ratio in different cultures. Glc, glucose (100 μM); C, sodium bicarbonate (2.5 mM); S, thiosulfate (1 mM). Error bars denote the SD of three replicates. Cellular C/N ratio, Glc: 4.13 ± 0.02; Glc+C: 4.13 ± 0.01; Glc+S: 4.04 ± 0.02; C+S: 4.08 ± 0.01; Glc+C+S: 4.08 ± 0.01.

Fig. S6. Transmission electron micrograph of negatively stained cells of *T. profunda* JLT2016 grown under different culture conditions at a magnification 2000× (bar, 2 μm) and a magnification of 6000× (bar, 0.5 μm). Glc, glucose (100 μM); C, sodium bicarbonate (2.5 mM); S, thiosulfate (1 mM).

Fig. S7. Image of *T. profunda* JLT2016 by confocal laser scanning. The excitation/emission wavelengths for the fluorophores were 400/490 nm and 570/602 nm. Blue indicates the extracellular substances and the red indicates cells.

Fig. S8. qRT-PCR studies of soxB and cbbL of *T. profunda* JLT2016 in the early stationary phase under different culture conditions.

Fig. S9. Determination of ATP, NADH and NADPH conditions in different cultures. Glc, glucose (100 μM); C, sodium bicarbonate (2.5 mM); S, thiosulfate (1 mM). Error

bars denote the SD of three replicates.

Fig. S10. Proteomics analyses of ribosomal proteins, EPS and PHB metabolisms related proteins under different culture conditions.

Fig. S11. Proteomics analyses of transporters, hydrogenase, sulfide quinone oxidoreductase and urease under different culture conditions.

Fig. S12. Phosphonate utilization (*phn*) gene clusters in two deep-sea roseobacter bacteria.

Fig. S13. Architectures of the genomic loci for subtypes of CRISPR–Cas systems of *P. abyssi* JLT2014 and *Rhodovulum* sp. NI22. The Cas operon consists of eight genes and the CRISPR sequences are located downstream from Cas2. *P. abyssi* JLT2014 and *Rhodovulum* sp. NI22 genomes contain two and one CRISPR arrays, respectively. Genomic context of the CRISPR arrays are listed on the table. One distinct base between direct repeats consensus of CRISPR arrays is shown in a highlight box. The numbers below the shaded areas represent sequence identity between two bacteria at the amino acid level.

Fig. S14. Pectin PULs in a plasmid of *T. profunda* JLT2016. **(A)** Genomic context of the pectin PULs. The numbers represent the sequence identity (%) between *T. profunda* JLT2016 and other bacteria. **(B)** Growth of *T. profunda* JLT2016 in an agar plate (left) and an agar plate with pectin (right); growth of *P. abyssi* JLT2014 in an agar plate with pectin (bottom).

Fig. S15. Multiple genome alignment performed using the Mauve progressive software and the chromosomes of *T. profunda* JLT2016 and *P. abyssi* JLT2014. *P. abyssi* JLT2014 is the reference for alignments and comparisons to *T. profunda* JLT2016. Lines link blocks with homology between two genomes. The colored bars inside the blocks are related to the level of sequence identity. Blocks anchored underneath the horizontal lines indicate regions with inverse orientations.

Fig. S16. Venn diagrams showing numbers of shared highly conserved genes families between the chromosomes and plasmids in the genomes of *T. profunda* JLT2016 and *P. abyssi* JLT2014 (>90% amino acid identity). Information regarding identical gene sequences of transposases, integrases and recombinases is listed in the figure.

Fig. S17. Genomic island (GI) prediction by different methods. Ring 1 (red) (from outside in) indicates GIs identified by multiple methods; ring 2 (blue) indicates GIs predicted using the IslandPath-DIMOB method; ring 3 (orange) indicates GIs predicted using the SIGI-HMM method; and the black line plot indicates the G+C content. Representative genes in GIs are listed in the upper left corner of the diagram. Details regarding the GIs of *T. profunda* JLT2016 and *P. abyssi* JLT2014 are listed in Supplementary Table S4 and Table S5, respectively.

CO_2 fixation

(DNA region (bp): 2563416-2575372, chromosome, *Thiobacimonas profunda* JLT2016)

IMG Gene ID	Gene name		*Pelagibaca abyssi* JLT2014	*Pelagibaca bermudensis* HTCC 2601	*Citreicella* sp. SE45	*Salipiger mucosus* DSM 16094	*Oceanicola* sp. MCTG156(1a)
2631100904	ribulose-phosphate 3-epimerase	cbbE	96	96	96	81	81
2631100905	probable Rubsico expression protein CbbX	cbbX	92	93	90	88	85
2631100906	ribulose-bisphosphate carboxylase small chain	cbbS	94	96	94	91	94
2631100907	ribulose-bisphosphate carboxylase large chain	cbbL	98	97	99	99	98
2631100908	fructose-bisphosphate aldolase, class II	cbbA	89	90	88	88	84
2631100909	phosphoglycerate kinase	cbbK	87	88			73
2631100910	glyceraldehyde 3-phosphate dehydrogenase	cbbG	90	92		87	87
2631100911	transketolase	cbbT	87	87	89	85	74
2631100912	phosphoribulokinase	cbbP	94	89	95	92	87
2631100913	fructose-1,6-bisphosphatase I	cbbF	90	94	87	81	68
2631100914	DNA-binding transcriptional regulator, LysR family	cbbR	90	89	87	82	56

Sulfur oxidation

(DNA region (bp): 512839-521700, chromosome, *Thiobacimonas profunda* JLT2016)

IMG Gene ID	Gene name		*Pelagibaca abyssi* JLT2014	*Pelagibaca bermudensis* HTCC2601	*Citreicella* sp. SE45	*Citreicella* sp. 357	*Salipiger mucosus* DSM 16094	*Oceanicola* sp. MCTG156(1a)	*Sagittula stellata* E-37
2631098920	sulfite dehydrogenase cytochrome subunit soxD	soxD	74	78	65	66	60	71	63
2631098921	sulfane dehydrogenase subunit soxC	soxC	93	92	89	84	80	93	86
2631098922	sulfur-oxidizing protein soxB	soxB	94	94	90	89	80	92	88
2631098923	sulfur-oxidizing protein soxA	soxA	88	89	83	78	74	85	80
2631098924	sulfur-oxidizing protein soxZ	soxZ	93	91	90	83	84	92	80
2631098925	sulfur-oxidizing protein soxY	soxY	94	93	78	84	69	87	64
2631098926	sulfur-oxidizing protein soxX	soxX	78	84	75	70	62	78	68
2631098927	thioredoxin-related protein	soxW	88	90	87	76	58	89	77
2631098928	cytochrome c-type biogenesis protein	soxE	89	90	90	84	72	88	88
2631098929	regulatory protein soxS	soxS	71	68	71	68	57	71	63
2631098930	ArsR family transcriptional regulator	soxR	89	86	84	83	72	85	87

Fig. S1

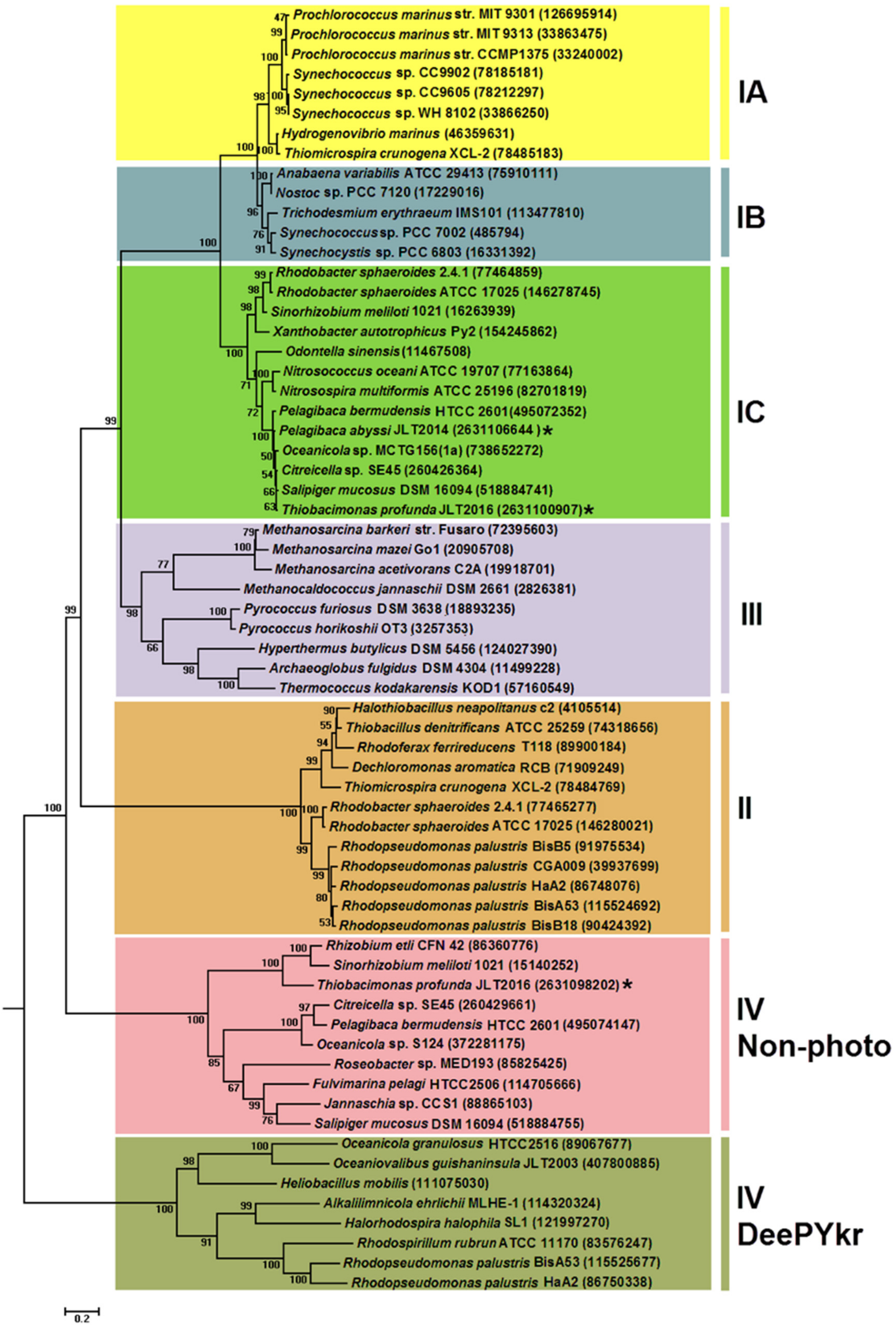

Prochlorococcus marinus str. MIT 9301 (126695914)
Prochlorococcus marinus str. MIT 9313 (33863475)
Prochlorococcus marinus str. CCMP1375 (33240002)
Synechococcus sp. CC9902 (78185181)
Synechococcus sp. CC9605 (78212297)
Synechococcus sp. WH 8102 (33866250)
Hydrogenovibrio marinus (46359631)
Thiomicrospira crunogena XCL-2 (78485183)
Anabaena variabilis ATCC 29413 (75910111)
Nostoc sp. PCC 7120 (17229016)
Trichodesmium erythraeum IMS101 (113477810)
Synechococcus sp. PCC 7002 (485794)
Synechocystis sp. PCC 6803 (16331392)
Rhodobacter sphaeroides 2.4.1 (77464859)
Rhodobacter sphaeroides ATCC 17025 (146278745)
Sinorhizobium meliloti 1021 (16263939)
Xanthobacter autotrophicus Py2 (154245862)
Odontella sinensis (11467508)
Nitrosococcus oceani ATCC 19707 (77163864)
Nitrosospira multiformis ATCC 25196 (82701819)
Pelagibaca bermudensis HTCC 2601(495072352)
Pelagibaca abyssi JLT2014 (2631106644) ★
Oceanicola sp. MCTG156(1a) (738652272)
Citreicella sp. SE45 (260426364)
Salipiger mucosus DSM 16094 (518884741)
Thiobacimonas profunda JLT2016 (2631100907) ★
Methanosarcina barkeri str. Fusaro (72395603)
Methanosarcina mazei Go1 (20905708)
Methanosarcina acetivorans C2A (19918701)
Methanocaldococcus jannaschii DSM 2661 (2826381)
Pyrococcus furiosus DSM 3638 (18893235)
Pyrococcus horikoshii OT3 (3257353)
Hyperthermus butylicus DSM 5456 (124027390)
Archaeoglobus fulgidus DSM 4304 (11499228)
Thermococcus kodakarensis KOD1 (57160549)
Halothiobacillus neapolitanus c2 (4105514)
Thiobacillus denitrificans ATCC 25259 (74318656)
Rhodoferax ferrireducens T118 (89900184)
Dechloromonas aromatica RCB (71909249)
Thiomicrospira crunogena XCL-2 (78484769)
Rhodobacter sphaeroides 2.4.1 (77465277)
Rhodobacter sphaeroides ATCC 17025 (146280021)
Rhodopseudomonas palustris BisB5 (91975534)
Rhodopseudomonas palustris CGA009 (39937699)
Rhodopseudomonas palustris HaA2 (86748076)
Rhodopseudomonas palustris BisA53 (115524692)
Rhodopseudomonas palustris BisB18 (90424392)
Rhizobium etli CFN 42 (86360776)
Sinorhizobium meliloti 1021 (15140252)
Thiobacimonas profunda JLT2016 (2631098202) ★
Citreicella sp. SE45 (260429661)
Pelagibaca bermudensis HTCC 2601 (495074147)
Oceanicola sp. S124 (372281175)
Roseobacter sp. MED193 (85825425)
Fulvimarina pelagi HTCC2506 (114705666)
Jannaschia sp. CCS1 (88865103)
Salipiger mucosus DSM 16094 (518884755)
Oceanicola granulosus HTCC2516 (89067677)
Oceaniovalibus guishaninsula JLT2003 (407800885)
Heliobacillus mobilis (111075030)
Alkalilimnicola ehrlichii MLHE-1 (114320324)
Halorhodospira halophila SL1 (121997270)
Rhodospirillum rubrum ATCC 11170 (83576247)
Rhodopseudomonas palustris BisA53 (115525677)
Rhodopseudomonas palustris HaA2 (86750338)
IA
IB
IC
III
II
IV
Non-photo
IV
DeePYkr
0.2

Fig. S2

Type IV secretion system
(DNA region (bp): 2668212-2686248, chromosome, *Thiobacimonas profunda* JLT2016, gene cluster1)

IMG Gene ID	Gene name		*Thiobacimonas profunda* JLT2016 (gene cluster2)	*Pelagibaca abyssi* JLT2014 (gene cluster1)	*Celeribacter baekdonensis* B30	*Oligotropha carboxidovorans* OM5	*Parvibaculum lavamentivorans* DS-1
2631101002	conjugative transfer signal peptidase TraF	traF	100	91	92	64	51
2631101003	uncharacterized conserved protein, DUF736 family		100	97	96	52	52
2631101004	type IV secretion system protein VirB1	virB1	91	84	87	65	65
2631101005	Type IV secretory pathway, VirD2 components (relaxase)	virD2	95	93	91	76	75
2631101006	type IV secretion system protein VirD4	virD4	97	97	97	81	83
2631101007	CopG domain-containing protein		96	96	97	81	73
2631101009	type IV secretion system protein VirB11	virB11	94	93	95	88	86
2631101010	type IV secretion system protein VirB2	virB2	100	96	95	76	75
2631101011	type IV secretion system protein VirB3	virB3	100	99	98	80	85
2631101012	type IV secretion system protein VirB4	virB4	100	95	95	83	82
2631101013	P-type conjugative transfer protein TrbJ	trbJ	100	97	97	74	76
2631101014	conjugative transfer region protein TrbK	trbK	100	88	87	56	54
2631101015	type IV secretion system protein TrbL	trbL	100	98	97	74	75
2631101016	type IV secretion system protein VirB5	virB5	100	99	99	86	83
2631101017	type IV secretion system protein VirB9	virB9	100	96	95	76	74
2631101018	type IV secretion system protein VirB10	virB10	100	96	95	70	71

Type IV secretion system*
(DNA region (bp): 13328-24440, plasmid pTPRO05, *Thiobacimonas profunda* JLT2016, gene cluster3)

IMG Gene ID	Gene name		*Pelagibaca abyssi* JLT2014, plasmid pPABY03 (gene cluster2)	*Thiobacimonas profunda* JLT2016, plasmid pTPRO06, (gene cluster4)	*Maritimibacter alkaliphilus* HTCC2654	*Dinoroseobacter shibae* DFL 12, plasmid pDSHI03
2631098061	type IV secretion system protein VirB6	virB6	82	23	89	81
2631098062	hypothetical protein		46		58	51
2631098063	hypothetical protein		46		80	
2631098064	type IV secretion system protein VirB11	virB11	87	47	85	86
2631098065	type IV secretion system protein VirB10	virB10	76	34	78	76
2631098066	type IV secretion system protein VirB9	virB9	88	37	87	81
2631098067	type IV secretion system protein VirB8	virB8	87	36	86	86
2631098068	type IV secretion system protein VirB5	virB5	94	35	93	90
2631098069	Transglycosylase SLT domain-containing protein		82	47	82	81
2631098070	hypothetical protein				61	60
2631098071	type IV secretion system protein VirB4	virB4	86	28	86	86
2631098072	type IV secretion system protein VirB3	virB3	91		91	91
2631098073	type IV secretion system protein VirB2	virB2	93	27	94	93
2631098074	type IV secretion system protein VirB1	virB1	69	42	68	68

*VirD2 (2631098052) and VirD4 (2631098053) genes are located near this gene cluster.

Fig. S3

H_2 utilization

(DNA region (bp):1748261-1766758, chromosome, *Thiobacimonas profunda* JLT2016)

IMG Gene ID	Gene name		*Pelagibaca bermudensis* HTCC2601	*Citreicella* sp. SE45	*Citreicella* sp. 357	*Sagittula stellata* E-37	*Roseovarius* sp. TM1035	*Rhodobacter sphaeroides* ATCC17025
2631100114	hydrogen sensor protein HupU	hupU	88	89	85	72	74	73
2631100115	hydrogen sensor protein HupV	hupV	81	82	77	68	67	64
2631100117	hydrogenase maturation protein HypF	hypF	74	77	71	72	68	65
2631100118	hydrogenase, small subunit	hupS	97	97	97	94	91	89
2631100119	hydrogenase, large subunit	hupL	91	92	89	92	86	88
2631100120	hydrogenase accessory protein HupE	hupE						58
2631100121	hydrogenase, cytochrome b subunit	hupC	88	87	87	71	63	65
2631100122	hydrogenase maturation protease HupD	hupD	85	87	84	70	72	67
2631100123	hydrogenase maturation chaperone HupF	hupF	72	72	70	59	59	58
2631100124	hydrogenase expression/formation protein HupG	hupG	89	85	80	63	68	68
2631100125	hydrogenase expression/formation protein HupH	hupH	80	79	73	65	64	60
2631100126	rubredoxin HupJ	hupJ	84	84	78	68	68	65
2631100127	hydrogenase expression/formation protein HupK	hupK	63	62	60	43	45	43
2631100128	hydrogenase nickel incorporation protein HypA	hypA	93	92	86	72	70	70
2631100129	hydrogenase nickel incorporation protein HypB	hypB	88	89	82	79	73	66
2631100130	hydrogenase transcriptional regulator HupR	hupR	85	86	89	63	61	58
2631100131	hydrogenase assembly chaperone HypC/HupF	hypC	86	86	80	75	72	73
2631100132	hydrogenase expression/formation protein HypD	hypD	91	92	89	82	84	81
2631100133	hydrogenase expression/formation protein HypE	hypE	91	88	88	77	74	73

Fig. S4

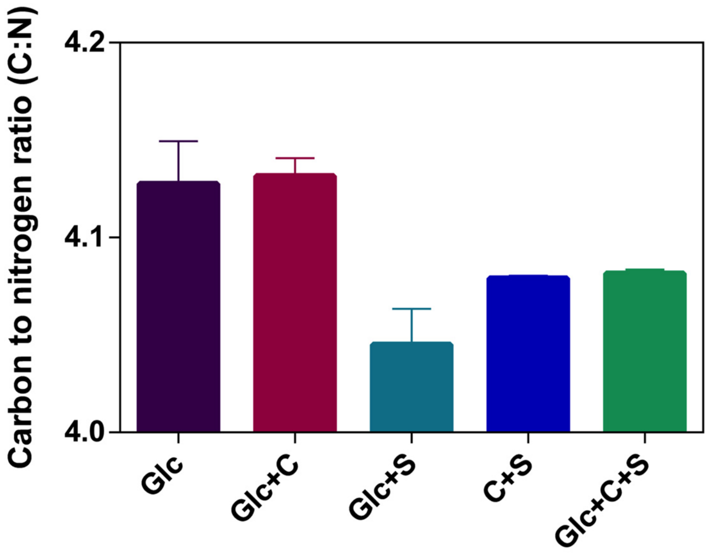

Fig. S5

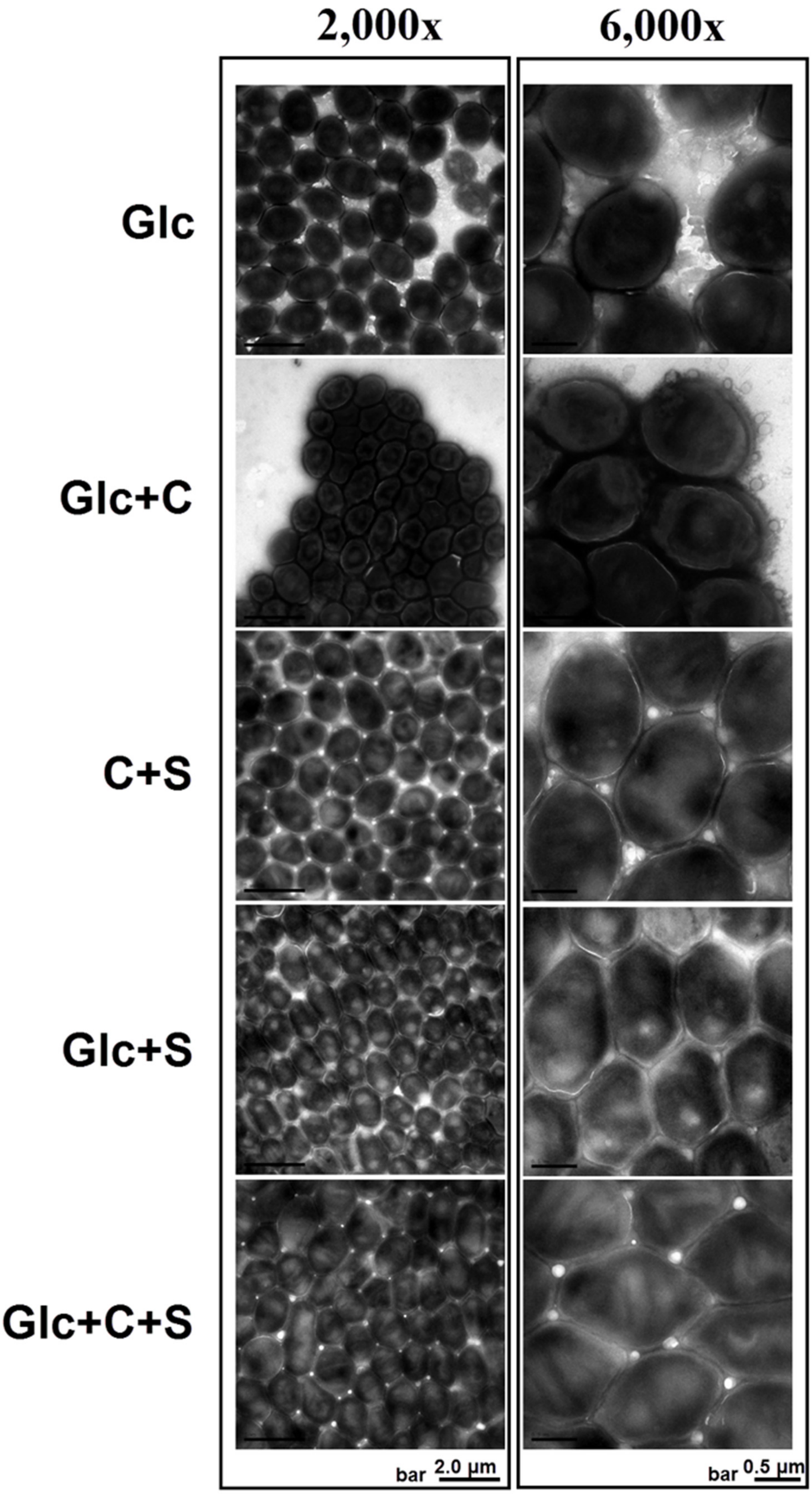

Fig. S6

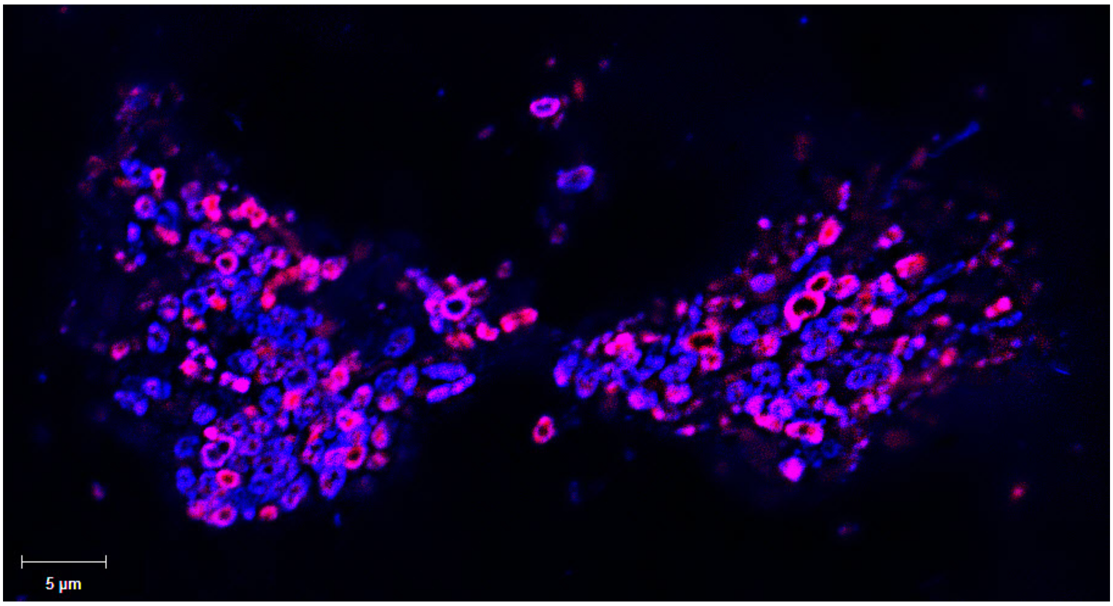

Fig. S7

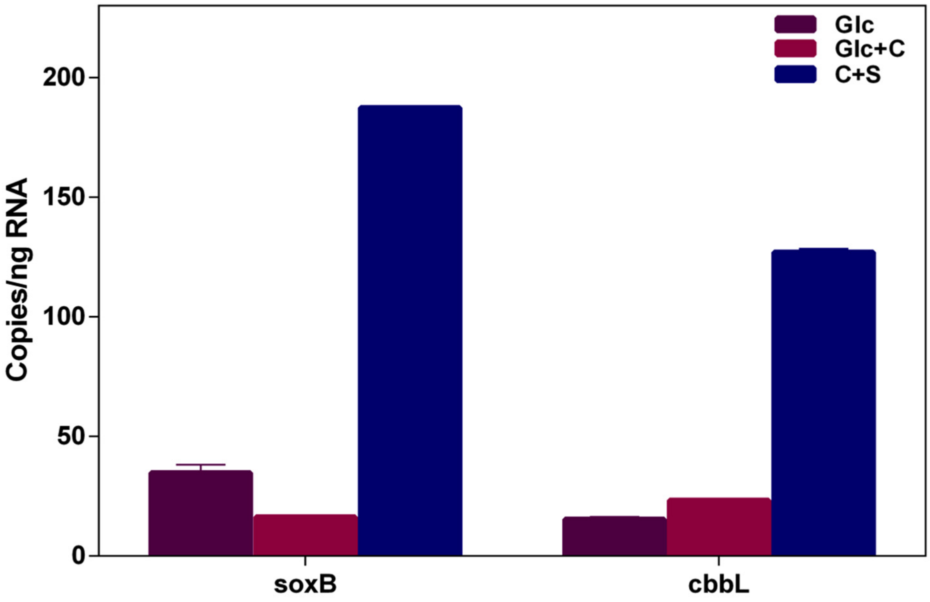

Fig. S8

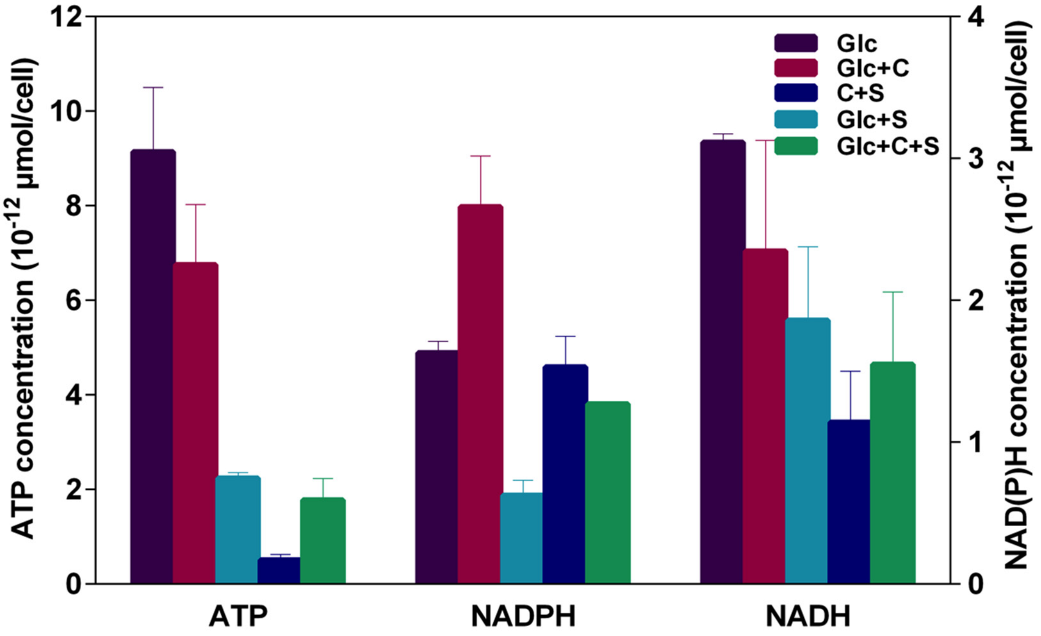
ATP concentration (10-12 μmol/cell)
NAD(P)H concentration (10-12 μmol/cell)
Glc
Glc+C
C+S
Glc+S
Glc+C+S
ATP
NADPH
NADH
0
2
4
6
8
10
12
1
3

Fig. S9

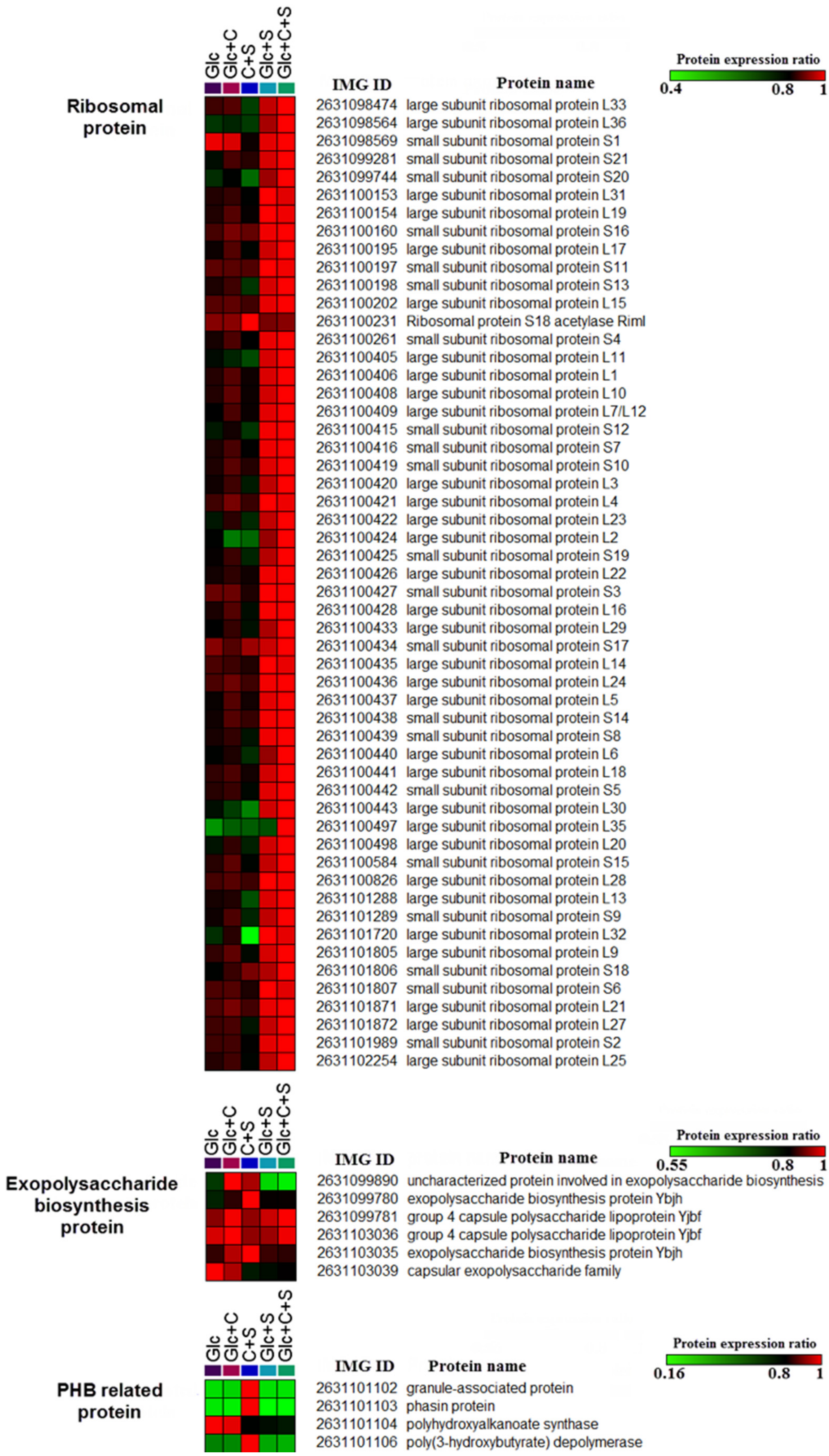
Glc
Glc+C
C+S
Glc+S
Glc+C+S
IMG ID
Protein name
Protein expression ratio
0.4
0.8
1
Ribosomal protein
2631098474 large subunit ribosomal protein L33
2631098564 large subunit ribosomal protein L36
2631098569 small subunit ribosomal protein S1
2631099281 small subunit ribosomal protein S21
2631099744 small subunit ribosomal protein S20
2631100153 large subunit ribosomal protein L31
2631100154 large subunit ribosomal protein L19
2631100160 small subunit ribosomal protein S16
2631100195 large subunit ribosomal protein L17
2631100197 small subunit ribosomal protein S11
2631100198 small subunit ribosomal protein S13
2631100202 large subunit ribosomal protein L15
2631100231 Ribosomal protein S18 acetylase RimI
2631100261 small subunit ribosomal protein S4
2631100405 large subunit ribosomal protein L11
2631100406 large subunit ribosomal protein L1
2631100408 large subunit ribosomal protein L10
2631100409 large subunit ribosomal protein L7/L12
2631100415 small subunit ribosomal protein S12
2631100416 small subunit ribosomal protein S7
2631100419 small subunit ribosomal protein S10
2631100420 large subunit ribosomal protein L3
2631100421 large subunit ribosomal protein L4
2631100422 large subunit ribosomal protein L23
2631100424 large subunit ribosomal protein L2
2631100425 small subunit ribosomal protein S19
2631100426 large subunit ribosomal protein L22
2631100427 small subunit ribosomal protein S3
2631100428 large subunit ribosomal protein L16
2631100433 large subunit ribosomal protein L29
2631100434 small subunit ribosomal protein S17
2631100435 large subunit ribosomal protein L14
2631100436 large subunit ribosomal protein L24
2631100437 large subunit ribosomal protein L5
2631100438 small subunit ribosomal protein S14
2631100439 small subunit ribosomal protein S8
2631100440 large subunit ribosomal protein L6
2631100441 large subunit ribosomal protein L18
2631100442 small subunit ribosomal protein S5
2631100443 large subunit ribosomal protein L30
2631100497 large subunit ribosomal protein L35
2631100498 large subunit ribosomal protein L20
2631100584 small subunit ribosomal protein S15
2631100826 large subunit ribosomal protein L28
2631101288 large subunit ribosomal protein L13
2631101289 small subunit ribosomal protein S9
2631101720 large subunit ribosomal protein L32
2631101805 large subunit ribosomal protein L9
2631101806 small subunit ribosomal protein S18
2631101807 small subunit ribosomal protein S6
2631101871 large subunit ribosomal protein L21
2631101872 large subunit ribosomal protein L27
2631101989 small subunit ribosomal protein S2
2631102254 large subunit ribosomal protein L25
Glc
Glc+C
C+S
Glc+S
Glc+C+S
IMG ID
Protein name
Protein expression ratio
0.55
0.8
1
Exopolysaccharide biosynthesis protein
2631099890 uncharacterized protein involved in exopolysaccharide biosynthesis
2631099780 exopolysaccharide biosynthesis protein Ybjh
2631099781 group 4 capsule polysaccharide lipoprotein Yjbf
2631103036 group 4 capsule polysaccharide lipoprotein Yjbf
2631103035 exopolysaccharide biosynthesis protein Ybjh
2631103039 capsular exopolysaccharide family
Glc
Glc+C
C+S
Glc+S
Glc+C+S
IMG ID
Protein name
Protein expression ratio
0.16
0.8
1
PHB related protein
2631101102 granule-associated protein
2631101103 phasin protein
2631101104 polyhydroxyalkanoate synthase
2631101106 poly(3-hydroxybutyrate) depolymerase

Fig. S10

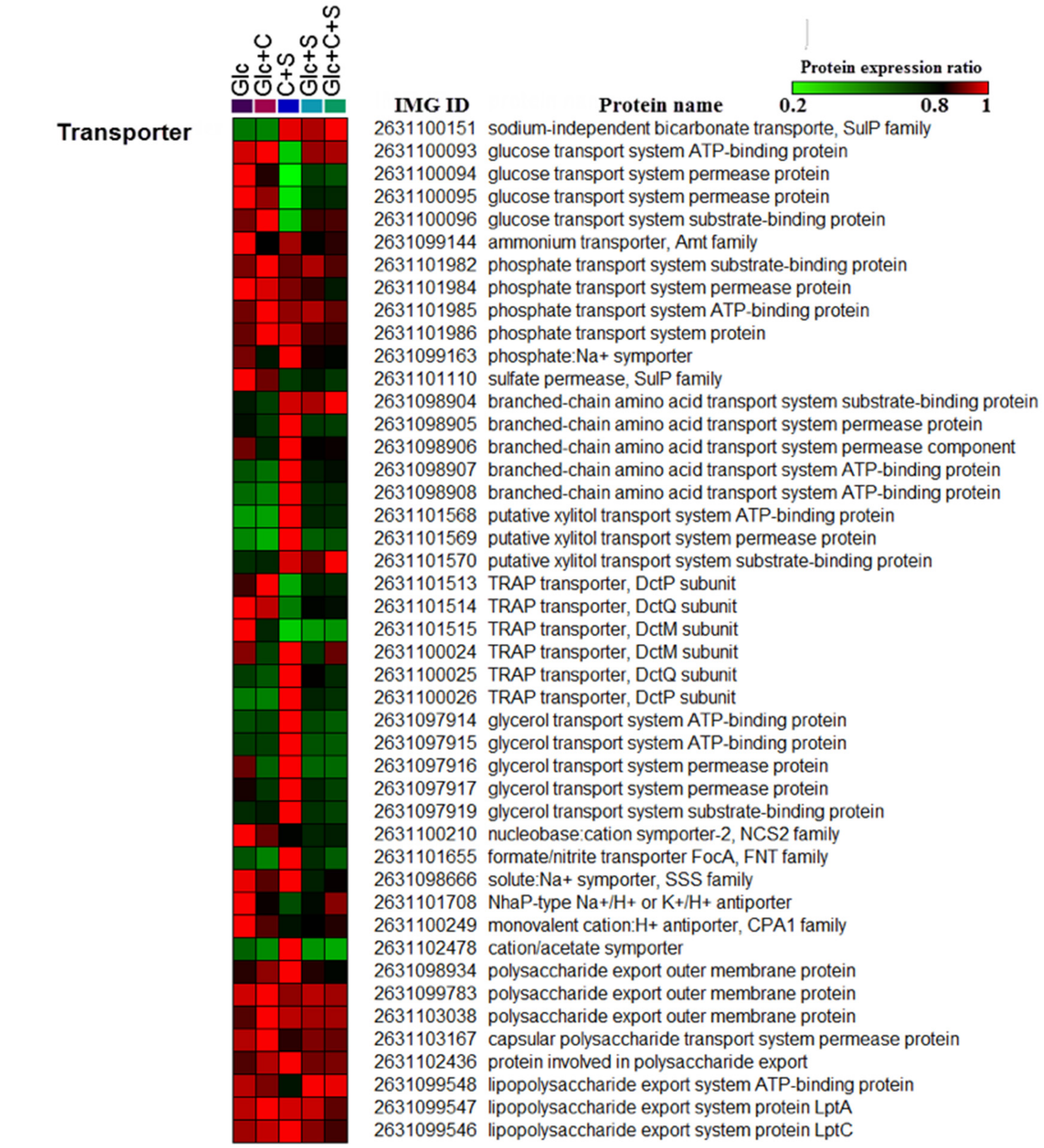
Glc
Glc+C
C+S
Glc+S
Glc+C+S
Protein expression ratio
0.2
0.8
1
IMG ID
Protein name
Transporter
2631100151 sodium-independent bicarbonate transporte, SulP family
2631100093 glucose transport system ATP-binding protein
2631100094 glucose transport system permease protein
2631100095 glucose transport system permease protein
2631100096 glucose transport system substrate-binding protein
2631099144 ammonium transporter, Amt family
2631101982 phosphate transport system substrate-binding protein
2631101984 phosphate transport system permease protein
2631101985 phosphate transport system ATP-binding protein
2631101986 phosphate transport system protein
2631099163 phosphate:Na+ symporter
2631101110 sulfate permease, SulP family
2631098904 branched-chain amino acid transport system substrate-binding protein
2631098905 branched-chain amino acid transport system permease protein
2631098906 branched-chain amino acid transport system permease component
2631098907 branched-chain amino acid transport system ATP-binding protein
2631098908 branched-chain amino acid transport system ATP-binding protein
2631101568 putative xylitol transport system ATP-binding protein
2631101569 putative xylitol transport system permease protein
2631101570 putative xylitol transport system substrate-binding protein
2631101513 TRAP transporter, DctP subunit
2631101514 TRAP transporter, DctQ subunit
2631101515 TRAP transporter, DctM subunit
2631100024 TRAP transporter, DctM subunit
2631100025 TRAP transporter, DctQ subunit
2631100026 TRAP transporter, DctP subunit
2631097914 glycerol transport system ATP-binding protein
2631097915 glycerol transport system ATP-binding protein
2631097916 glycerol transport system permease protein
2631097917 glycerol transport system permease protein
2631097919 glycerol transport system substrate-binding protein
2631100210 nucleobase:cation symporter-2, NCS2 family
2631101655 formate/nitrite transporter FocA, FNT family
2631098666 solute:Na+ symporter, SSS family
2631101708 NhaP-type Na+/H+ or K+/H+ antiporter
2631100249 monovalent cation:H+ antiporter, CPA1 family
2631102478 cation/acetate symporter
2631098934 polysaccharide export outer membrane protein
2631099783 polysaccharide export outer membrane protein
2631103038 polysaccharide export outer membrane protein
2631103167 capsular polysaccharide transport system permease protein
2631102436 protein involved in polysaccharide export
2631099548 lipopolysaccharide export system ATP-binding protein
2631099547 lipopolysaccharide export system protein LptA
2631099546 lipopolysaccharide export system protein LptC

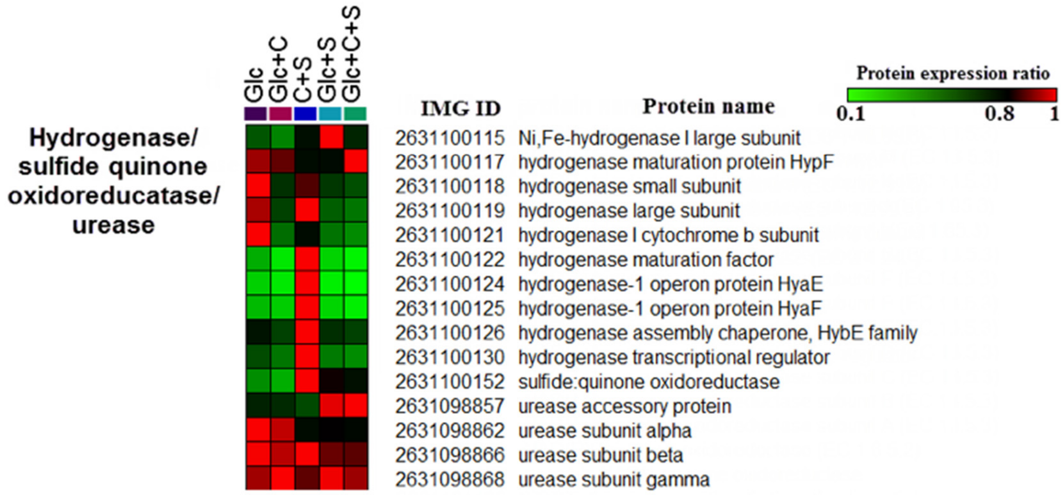
Glc
Glc+C
C+S
Glc+S
Glc+C+S
Protein expression ratio
0.1
0.8
1
IMG ID
Protein name
Hydrogenase/
sulfide quinone
oxidoreducatase/
urease
2631100115 Ni,Fe-hydrogenase I large subunit
2631100117 hydrogenase maturation protein HypF
2631100118 hydrogenase small subunit
2631100119 hydrogenase large subunit
2631100121 hydrogenase I cytochrome b subunit
2631100122 hydrogenase maturation factor
2631100124 hydrogenase-1 operon protein HyaE
2631100125 hydrogenase-1 operon protein HyaF
2631100126 hydrogenase assembly chaperone, HybE family
2631100130 hydrogenase transcriptional regulator
2631100152 sulfide:quinone oxidoreductase
2631098857 urease accessory protein
2631098862 urease subunit alpha
2631098866 urease subunit beta
2631098868 urease subunit gamma

Fig. S11

Phosphonate utilization
(DNA region (bp): 17992-29898, plasmid pTPRO01, *Thiobacimonas profunda* JLT2016)

IMG Gene ID	Gene name		*Pelagibaca abyssi* JLT2014 (gene culster1)	*Pelagibaca abyssi* JLT2014 (gene cluster2)	*Pelagibaca bermudensis* HTCC 2601
2631098178	phosphonate transport system ATP-binding protein	phnC			83
2631098179	phosphonate transport system substrate-binding protein	phnD			74
2631098180	phosphonate transport system permease protein	phnE			76
2631098181	phosphonate transport system permease protein	phnE			70
2631098183	phosphonate utilization regulatory gene	phnF	57	31	55
2631098184	ribophosphonate triphosphate synthase subunit PhnG	phnG	73	28	72
2631098185	ribophosphonate triphosphate synthase subunit PhnH	phnH	60	29	59
2631098186	ribophosphonate triphosphate synthase subuit PhnI	phnI	78	40	80
2631098187	rhosphoribosyl phosphonate carbon-phosphorus lyase	phnJ	84	49	86
2631098188	putative phosphonate transport system ATP-binding protein	phnK	85	49	88
2631098189	ribophosphonate triphosphate synthase subunit PhnL	phnL	76	41	75
2631098190	ribose 1,5-bisphosphokinase	phnN	53		61
2631098192	ribophosphonate triphosphate hydrolase	phnM	64	41	65

Fig. S12

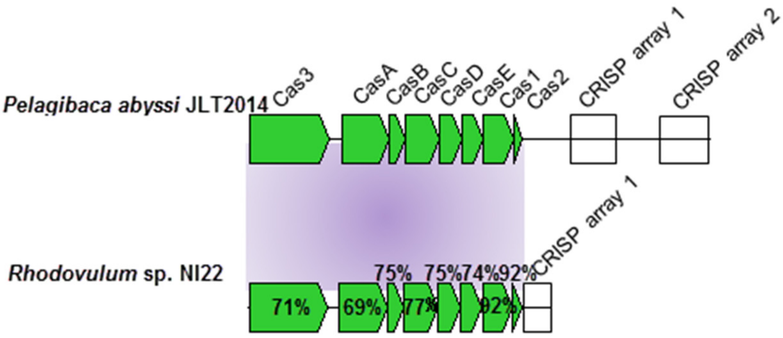

		Start position	End position	length	Spacers count	Direct repeats consensus
Pelagibaca abyssi JLT2014	CRISP array 1	2040159	2041469	1310	21	CCGTTCCCCGCTCGCGCGGGGATCAACCG
	CRISP array 2	2042909	2044340	1431	23	CCGTTCCCCGCTCGCGCGGGGATCAACCG
Rhodovulum sp. NI22	CRISP array 1	9831	10530	699	11	CCGTTCCCCGCCCGCGCGGGGATCAACCG

Fig. S13

A

Pectin utilization locus
(DNA region (bp): 92115-114107, plasmid pTPRO01, *Thiobacimonas profunda* JLT2016)

IMG Gene ID	Gene name	*Pelagibaca bermudensis* HTCC 2601	*Salipiger mucosus* DSM 16094	*Rhizobium lupini* HPC(L)	*Agrobacterium* sp. H13-3
2631098248	pectin degradation protein	83	92	69	69
2631098249	AraC-type DNA-binding protein	93	73	61	60
2631098250	oligogalacturonide transport system permease protein	92	92	83	82
2631098251	oligogalacturonide transport system permease protein	91		85	84
2631098252	oligogalacturonide transport system ATP-binding protein	88	92	71	71
2631098253	oligogalacturonide transport system substrate-binding protein	77	89	67	67
2631098254	polygalacturonase	77	78	59	60
2631098255	unsaturated rhamnogalacturonyl hydrolase	83	83	65	66
2631098256	D-galactarolactone isomerase	92	80	57	57
2631098257	D-galactarolactone cycloisomerase	96	92	75	74
2631098258	5-dehydro-4-deoxyglucarate dehydratase	82	92	81	80
2631098259	NADP-dependent aldehyde dehydrogenase	82	88		
2631098260	uronate dehydrogenase	85	87	66	65
2631098261	TRAP transporter, DctM subunit	97	95	52	52
2631098262	TRAP transporter, small permease component	80	80	36	34
2631098263	TRAP transporter solute receptor, DctP family	90	92	45	44
2631098267	2-deoxy-D-gluconate 3-dehydrogenase	88	87	72	72
2631098268	4-deoxy-L-threo-5-hexosulose-uronate ketol-isomerase	93	82	77	77

B

***T. profunda* JLT2016**

***P. abyssi* JLT2014**

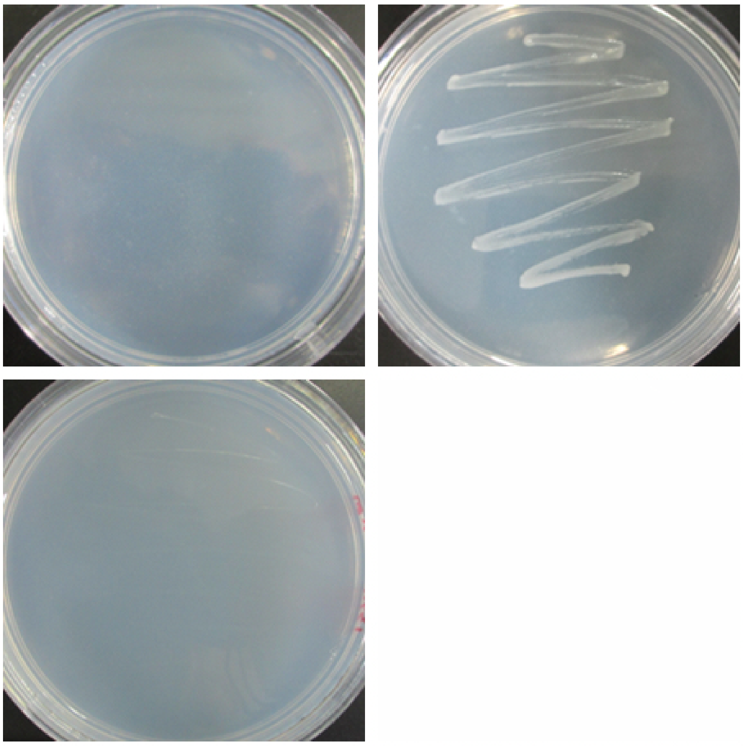

Fig. S14

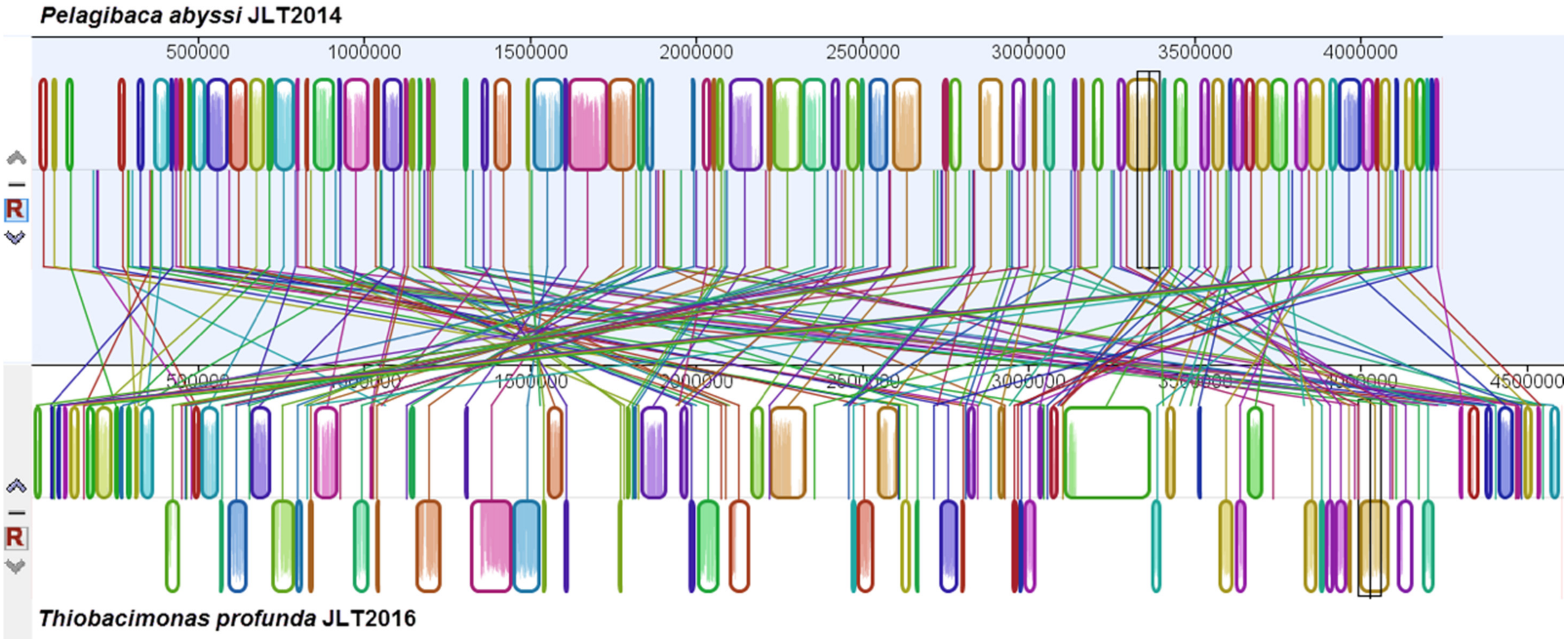
Pelagibaca abyssi JLT2014
500000
1000000
1500000
2000000
2500000
3000000
3500000
4000000
4500000
R
R
Thiobacimonas profunda JLT2016

Fig. S15

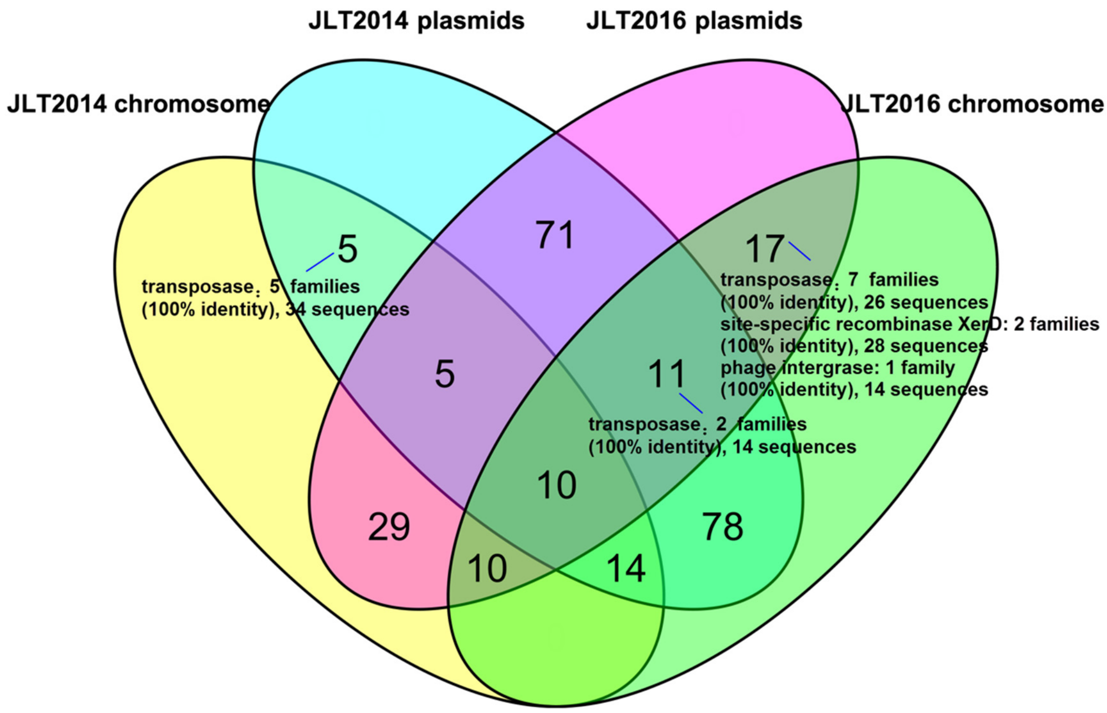
JLT2014 plasmids
JLT2016 plasmids
JLT2014 chromosome
JLT2016 chromosome
5
transposase：5 families
(100% identity), 34 sequences
71
17
transposase：7 families
(100% identity), 26 sequences
site-specific recombinase XerD: 2 families
(100% identity), 28 sequences
phage intergrase: 1 family
(100% identity), 14 sequences
5
11
transposase：2 families
(100% identity), 14 sequences
10
29
78
10
14

Fig. S16

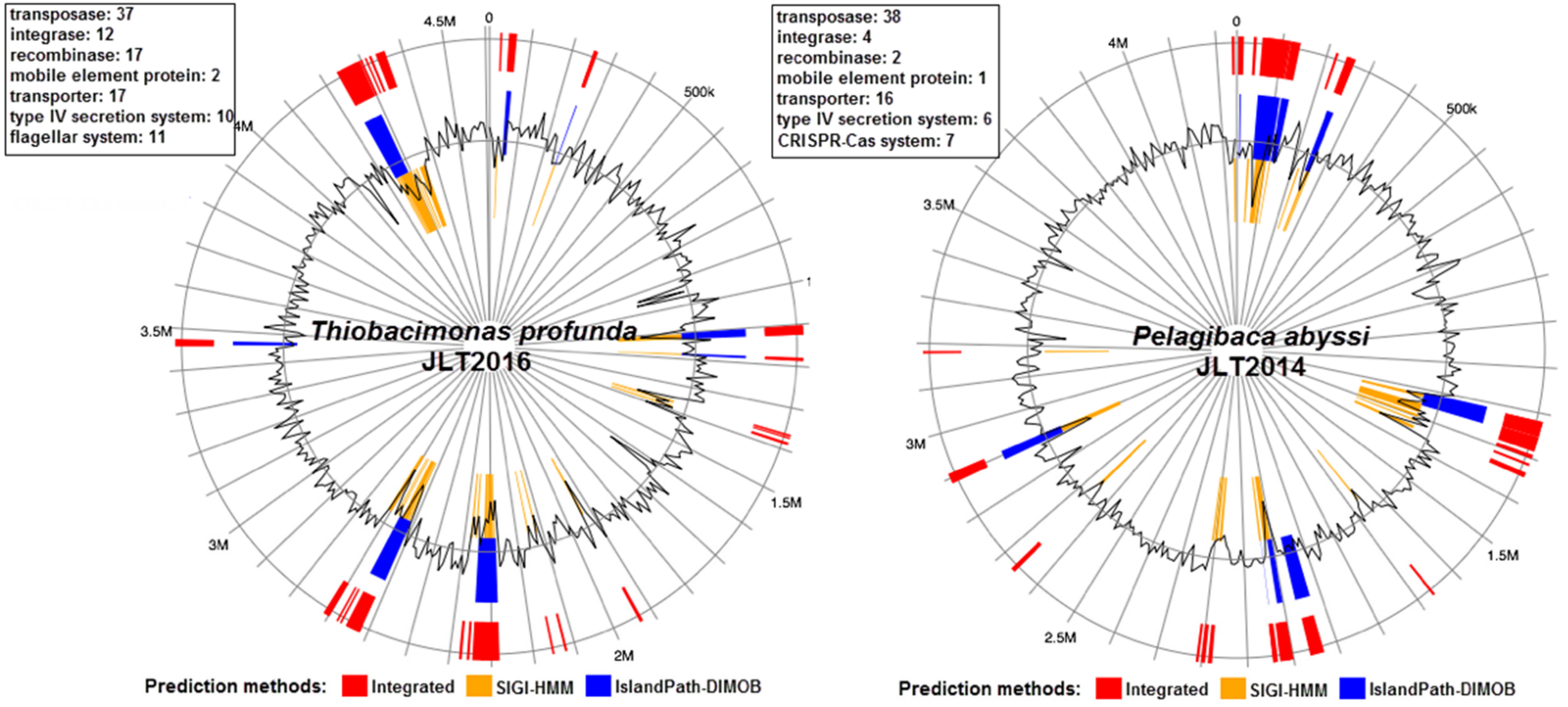
transposase: 37
integrase: 12
recombinase: 17
mobile element protein: 2
transporter: 17
type IV secretion system: 10
flagellar system: 11
Thiobacimonas profunda
JLT2016
0
500k
1.5M
2M
3M
3.5M
4M
4.5M
Prediction methods: Integrated SIGI-HMM IslandPath-DIMOB
transposase: 38
integrase: 4
recombinase: 2
mobile element protein: 1
transporter: 16
type IV secretion system: 6
CRISPR-Cas system: 7
Pelagibaca abyssi
JLT2014
0
500k
1.5M
2.5M
3M
3.5M
4M
Prediction methods: Integrated SIGI-HMM IslandPath-DIMOB

Fig. S17
